# Large Language Model Agent for Modular Task Execution in Drug Discovery

**DOI:** 10.1021/acs.jcim.5c02454

**Published:** 2026-02-09

**Authors:** Janghoon Ock, Radheesh Sharma Meda, Srivathsan Badrinarayanan, Neha S. Aluru, Achuth Chandrasekhar, Amir Barati Farimani

**Affiliations:** † Department of Chemical Engineering, 6612 Carnegie Mellon University, 5000 Forbes Avenue, Pittsburgh, Pennsylvania 15213, United States; ‡ Department of Chemical and Biomolecular Engineering, 14719 University of Nebraska—Lincoln, Lincoln, Nebraska 68588, United States; ¶ School of Engineering Medicine, 14736 Texas A&M University, Houston, Texas 77030, United States; § Department of Material Science and Engineering, 6612 Carnegie Mellon University, 5000 Forbes Avenue, Pittsburgh, Pennsylvania 15213, United States; ∥ Department of Mechanical Engineering, 6612 Carnegie Mellon University, 5000 Forbes Avenue, Pittsburgh, Pennsylvania 15213, United States

## Abstract

We present a modular framework powered by large language models (LLMs) that automates and streamlines key tasks across the early stage computational drug discovery pipeline. By combining LLM reasoning with domain-specific tools, the framework performs biomedical data retrieval, literature-grounded question answering via retrieval-augmented generation, molecular generation, multiproperty prediction, property-aware molecular refinement, and 3D protein–ligand structure generation. The agent autonomously retrieves relevant biomolecular information, including FASTA sequences, SMILES representations, and literature, and answers mechanistic questions with improved contextual accuracy compared to standard LLMs. It then generates chemically diverse seed molecules and predicted 75 properties, including ADMET-related and general physicochemical descriptors, which guids iterative molecular refinement. Across two refinement rounds, the number of molecules with QED >0.6 increased from 34 to 55. The number of molecules satisfying empirical drug-likeness filters also rose; for example, compliance with the Ghose filter increased from 32 to 55 within a pool of 100 molecules. The framework also employed Boltz-2 to generate 3D protein–ligand complexes and provide rapid binding affinity estimates for candidate compounds. These results demonstrate that the approach effectively supports molecular screening, prioritization, and structure evaluation. Its modular design enables flexible integration of evolving tools and models, providing a scalable foundation for AI-assisted therapeutic discovery.

## Graphical abstract

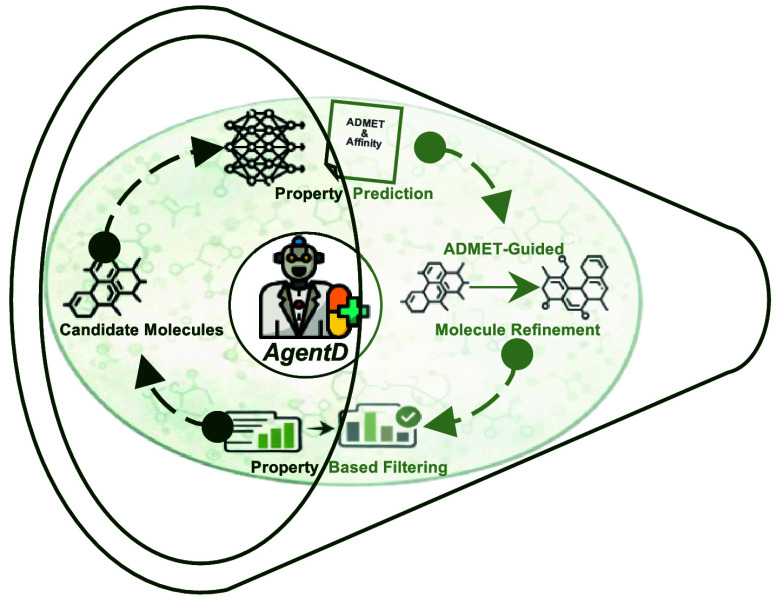

## Introduction

The discovery of drug-like molecules is fundamental to therapeutic innovation and the advancement of public health. However, identifying viable candidates is a highly complex and resource-intensive process. It requires navigating an enormous chemical space, integrating heterogeneous biological and chemical data sources, and conducting iterative experimental validation. Empirically, drug development spans 10–15 years and can cost $1.5–2.5 billion per approved therapy. Much of this burden arises from attrition in Phase II and III clinical trials, where insufficient efficacy or safety issues often lead to late-stage failures.^1–3^ A key strategy to reduce this attrition is to identify high-quality candidates as early as possible, thereby improving the likelihood of clinical success. At the same time, the chemical search space is astronomically large, with estimates of more than 10^60^ drug-like molecules, making it impossible to explore effectively with traditional trial-and-error approaches.^4,5^

To mitigate these challenges, computational approaches have become essential in early stage drug development. Methods such as molecular docking,^6–8^ quantitative structure–activity relationship (QSAR) modeling,^9–11^ and molecular dynamics simulations^12–14^ have significantly improved the speed and accuracy of compound screening and optimization. More recently, machine learning (ML) techniques have expanded this toolkit by enabling predictive modeling of pharmacokinetic and pharmacodynamic properties, de novo molecular generation, and multitask learning across diverse biomedical tasks.^15–18^ AlphaFold has achieved high-accuracy protein structure prediction.^19–21^ In parallel, generative models such as Variational Autoencoders (VAEs), Generative Adversarial Networks (GANs), and diffusion models (e.g., JT-VAE, MolGAN, MolDiff) enable de novo or target-specific molecular design.^22–24^ Also, Graph Neural Networks (GNNs) and transformer-based models improve property prediction.^25–28^ Collectively, these methods increase the scalability and automation potential of modern drug discovery workflows.

Despite this progress, most computational methods remain task-specific and require manual orchestration by domain experts. Drug discovery is inherently a multistep, interdependent process that requires seamless integration across diverse tasks.^29–31^ This fragmented implementation limits the scalability and efficiency of current pipelines. As the demand for faster and more cost-effective drug screening grows, there is an urgent need for unified, intelligent platforms capable of autonomously coordinating these tasks while supporting expert decision-making.

Large Language Models (LLMs) offer a compelling solution to this need. Trained on massive corpora of natural language data, LLMs exhibit strong reasoning capabilities and domain-agnostic knowledge. When augmented with external tools, such as domain-specific plugins, APIs, and software libraries, LLM-based agents can overcome the limitations of general-purpose language models and act as interpretable, flexible controllers of scientific workflows.^32,33^

LLM agents have recently been applied to automate diverse aspects of scientific discovery, including experimental design, material synthesis planning, and data analysis.^34–38^ For instance, the dZiner framework leverages LLM agents for molecular design through iterative reasoning and structure-based optimization.^39^ CACTUS^40^ demonstrated the use of an LLM to wrap cheminformatics utilities for evaluating properties such as Mwt, LogP, TPSA, and drug-likeness filters, providing a practical but narrowly scoped toolset. In contrast, SciToolAgent^41^ introduced a knowledge-graph–driven orchestration layer that allows an LLM to select from hundreds of scientific tools across domains ranging from biological to materials, illustrating scalability but lacking a dedicated workflow for therapeutic design. Building on these directions, DrugPilot^42^ focuses on orchestrating multistage discovery workflows using parametrized reasoning, primarily demonstrated on established model-zoo tasks and benchmark data sets rather than end-to-end molecular design. A more detailed overview of the comparative landscape of agentic frameworks is provided in Supporting Information Section 9.

We introduce AgentD, an LLM-powered agent framework designed to support and streamline the drug discovery pipeline. The agent performs a wide range of important tasks, including biomedical data retrieval from structured databases and unstructured web sources, answering domain-specific scientific queries, generating seed molecule libraries via SMILES-based generative models, predicting a broad spectrum of drug-relevant properties, refining molecular representations to improve drug-likeness, and generating 3D molecular structures for downstream analysis. Our results demonstrate that this agent-driven framework streamlines early phase drug discovery and serves as a flexible foundation for scalable, AI-assisted therapeutic development. Its modular architecture allows for continual improvement as more advanced tools and models become available.

## Agentic Workflow

### Task Modules

AgentD performs six essential tasks across the drug discovery pipeline, as illustrated in Figure 1a. From a code-design perspective, the agent integrates LLMs sourced from OpenAI, Anthropic, and DeepSeek. These models function both as the core reasoning module and as the natural-language interaction layer. In the present implementation, AgentD is primarily developed and executed using OpenAI’s GPT-4o as the main language model. By integrating domain-specific tools and databases, AgentD coordinates a wide range of activities - from data retrieval and molecular generation to property evaluation and structure prediction. The primary tool components supporting each task module are summarized in Table 1.

**Figure 1:**
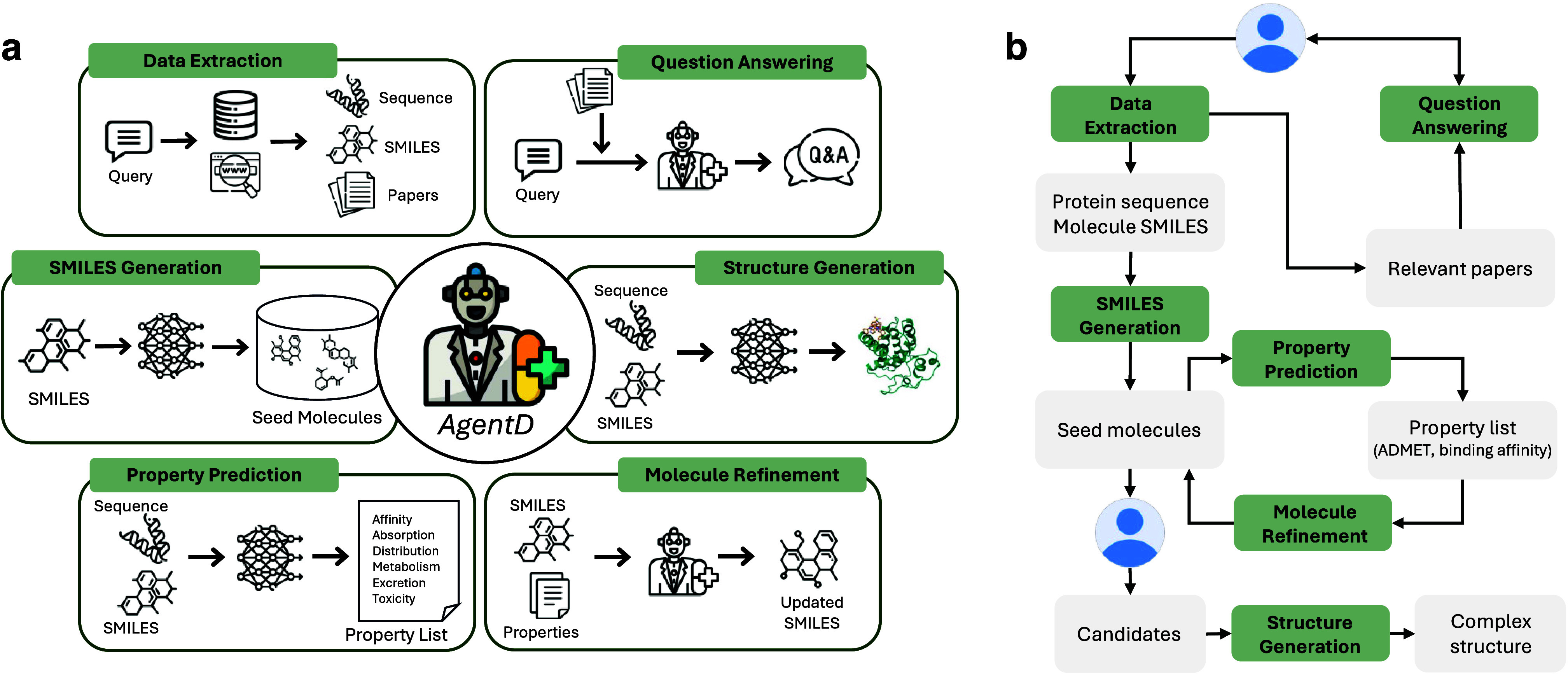
AgentD overview. (a) Task modules supported by AgentD. In question-answering and molecular-refinement tasks, outputs are generated directly by the language model, while other tasks rely on external computational tools for final results. (b) Workflow illustrating how each task module contributes to the overall drug discovery pipeline.

**Table 1:** Key External Tools Integrated into Each Task Module within the AgentD Drug Discovery Pipeline

**Task Module**	**Primary Tool Components**
Data Extraction	UniProt,^43^ ChEMBL,^44^ Serper,^52^ Semantic Scholar APIs
Question Answering	RAG, LLM internal knowledge and reasoning
SMILES Generation	REINVENT,^46^ Mol2Mol^47^
Property Prediction	DeepPK API,^48^ BAPULM^49^
Molecule Refinement	LLM internal knowledge and reasoning^a^
Structure Generation	Boltz-2 ^51^

a Adapted from the dZiner framework.^39^

#### Data Extraction

AgentD is capable of retrieving biomedical information from both structured and unstructured sources. Given a query, such as identifying known drugs associated with a specific protein–disease context, the agent retrieves the protein’s FASTA sequence from UniProt,^43^ searches for relevant drug names on the web, and extracts corresponding SMILES representations from ChEMBL.^44^ Additionally, AgentD autonomously constructs keyword-based queries to download relevant open-access scientific literature from Semantic Scholar, providing a broader context for downstream reasoning and decision-making.

#### Question Answering

In therapeutic applications, high accuracy and mechanistic specificity are critical. Generic responses that only appear plausible are inadequate for addressing domain-specific questions relevant to drug discovery and biomedical research. To handle domain-specific scientific queries, AgentD employs a retrieval-augmented generation (RAG) strategy. By grounding its responses in literature obtained during the data extraction phase, the agent provides context-aware and evidence-based answers.

#### SMILES Generation

Constructing a diverse and chemically relevant seed molecule pool is essential for effective early stage virtual screening. AgentD generates seed molecules in SMILES format using external generative models. In this study, we incorporate two such models: REINVENT,^45,46^ which supports de novo molecule generation without requiring input SMILES, and Mol2Mol,^47^ which performs conditional generation to produce molecules structurally similar to a given input SMILES. This dual capability enables both exploration and exploitation in the molecular search space.

#### Property Prediction

For each candidate molecule, AgentD predicts key pharmacologically relevant properties, including ADMET (absorption, distribution, metabolism, excretion, and toxicity) profiles and binding affinity (e.g., *pK*_*d*_). ADMET prediction is performed using the Deep-PK API,^48^ which accepts SMILES strings as input. For binding affinity estimation, the BAPULM model^49^ is used, which operates on both the SMILES and the protein’s FASTA sequence. These predictions help prioritize compounds based on both efficacy and safety.

#### Molecule Refinement

Based on the predicted properties, AgentD can identify molecular shortcomings such as toxicity or poor permeability, and propose targeted structural modifications to improve attributes like solubility and metabolic stability. This SMILES refinement is carried out solely through the LLM’s internal reasoning and built-in chemical knowledge, without additional model-based tools.^39^ This task is carried out on top of the property prediction stage.

#### Structure Generation

AgentD can generate 3D structures of protein–ligand complexes using Boltz-2 as an external tool.^50,51^ This process produces candidate complex structures along with associated binding metrics such as IC_50_ values and inhibitor probability. Since these structures and affinity outputs are model-derived, downstream physical validation (e.g., MD-based simulations and free-energy calculations) is required before drawing the final conclusions about binding stability or potency.

### Workflow

All six task modules in AgentD are designed to support key components of the drug discovery pipeline, as illustrated in Figure 1b. The process begins with a user-provided query, such as identifying drug molecules that target a specific disease-related protein. Through the data extraction module, AgentD retrieves the protein’s FASTA sequence from UniProt and identifies known drugs using sources such as ChEMBL and Google search APIs. In this study, we demonstrate the workflow using three representative therapeutic targets: BCL-2 for lymphocytic leukemia, JAK-2 for myelofibrosis, and thrombin for thrombosis. The BCL-2 case is discussed in detail in the main manuscript, while results for the other two targets are provided in Supporting Information Sections 5 and 6.

The AgentD framework checks whether known drug molecules exist for a given target or disease. If available, these molecules serve as starting points for subsequent molecular exploration. Because AgentD includes a de novo generation module, the workflow can still proceed even when no such molecules are found. The SMILES generation task builds a chemically diverse library of candidate molecules using generative models. These molecules are then evaluated using property prediction tools to estimate key pharmacological characteristics such as ADMET profiles, general physicochemical properties, and binding affinity. Based on these predicted properties, AgentD proposes SMILES-level modifications to improve attributes like solubility, toxicity, and metabolic stability. These refinements rely on the language model’s internal reasoning rather than external optimization tools and help enrich the candidate pool.

Following refinement, domain-specific criteria can be applied by the user to select promising compounds. For these, the structure generation task creates 3D protein–ligand complexes using the ligand SMILES and target protein sequence. This enables more detailed downstream analyses. The structure generation step also outputs auxiliary metrics like predicted IC_50_ and inhibitor probability as proxies for binding strength. Although these estimates from Boltz-2 provide useful initial guidance, downstream physical validation via MD simulations is required to assess binding stability and interaction fidelity. Additional uncertainty information, including Boltz-2–derived affinity estimates, is provided in Supporting Information Section 10.

Throughout the workflow, users may request clarification or validation of scientific concepts. The question answering module, implemented using RAG, supports this by leveraging literature collected during the data extraction phase. This functionality serves both to enhance the pipeline and to address user-specified scientific queries on demand.

## Results

### Data Extraction

AgentD integrates web search capabilities and database API access as tool modules for extracting protein and molecule data. When provided with a user query specifying a target protein and associated disease, the agent is instructed to (i) retrieve the protein’s FASTA sequence, (ii) identify existing drugs relevant to the specified target–disease context, and (iii) download related open-access scientific literature. The agent was tested on example queries including BCL-2 for lymphocytic leukemia, JAK2 for myelofibrosis, and thrombin for thrombosis. The results, summarized in Figure 2, show that the agent successfully retrieved key molecular data, such as identifying venetoclax as a BCL-2 inhibitor.

**Figure 2:**
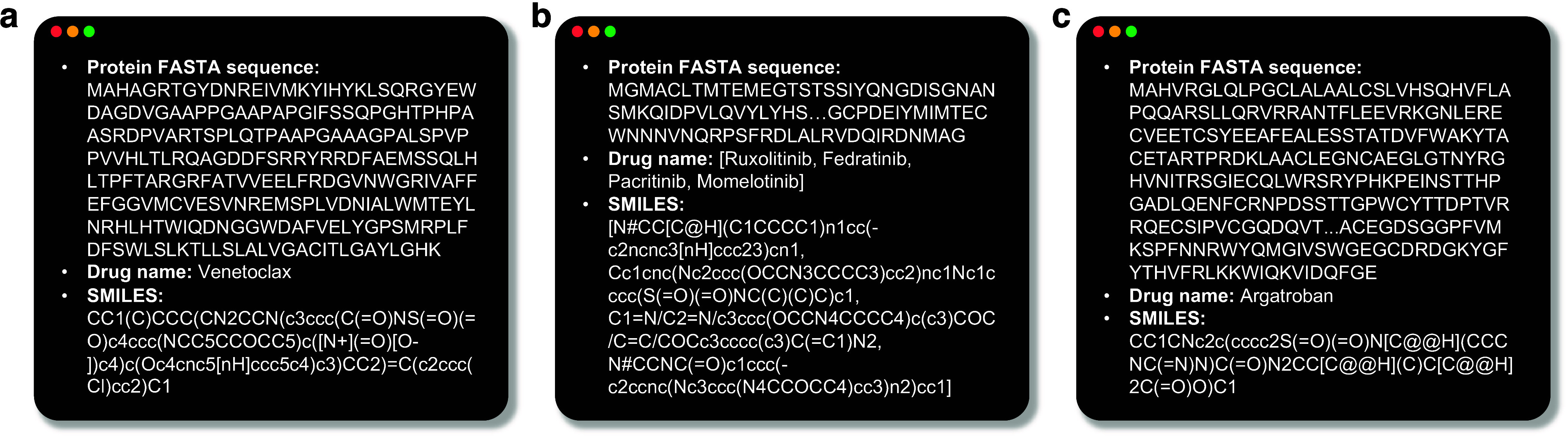
Examples of data extraction. Given a user query specifying a target protein and associated disease, AgentD retrieves the protein’s FASTA sequence and identifies known drug molecules relevant to the query. (a) BCL-2 for lymphocytic leukemia, (b) JAK-2 for myelofibrosis, and (c) thrombin for thrombosis.

The extracted information is used in several downstream modules. The retrieved SMILES is used to initialize molecule generation models for building a seed library. Both the SMILES and protein sequence serve as inputs to the property prediction and 3D structure generation components. Additionally, the downloaded documents are embedded into a vector database, enabling context-aware retrieval during the agent’s question answering task.

### Domain-Specific Question Answering

To address the limitations of generic, unsupported answers in biomedical contexts, AgentD employs RAG to ground its responses in domain-specific literature. During the data extraction phase, the agent formulates keyword-based queries from the target protein and disease, retrieves open-access papers via Semantic Scholar, and stores them in a vector database for later retrieval during question answering.

We evaluated this capability using a representative study by Weller et al., which describes how venetoclax activates the integrated stress response (ISR), leading to NOXA upregulation and MCL-1 inhibition.^53^ Based on this paper, we formulated domain-specific questions and compared the responses generated by AgentD with RAG to those from the vanilla GPT-4o model (Figure 3).

**Figure 3:**
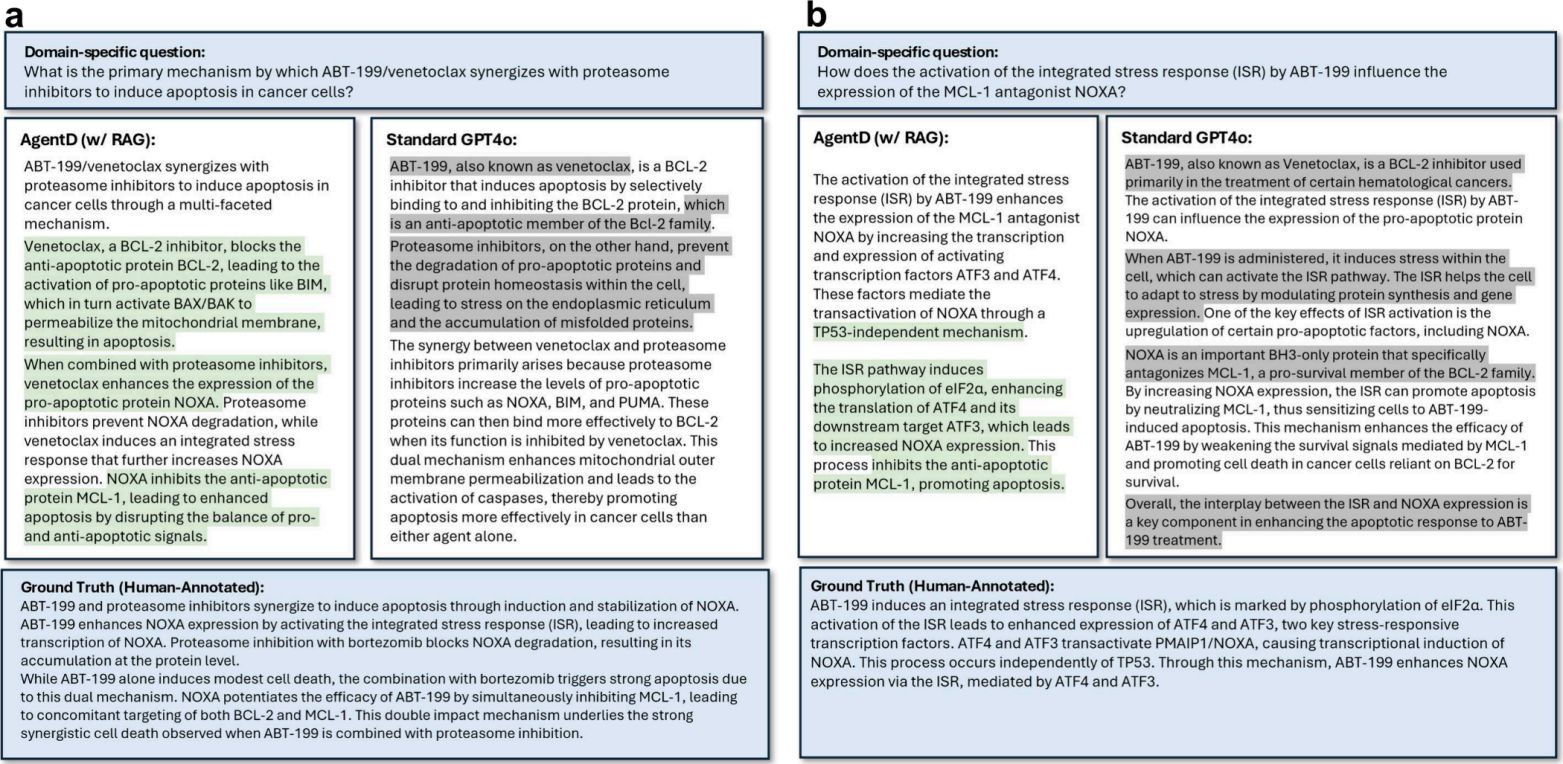
Comparison of question-answering performance between AgentD with RAG and the Vanilla GPT-4o model. Green highlights indicate grounded, context-relevant information supported by the reference paper, while gray highlights denote generic content not directly related to the question. (a) Mechanism of ABT-199/venetoclax synergy with proteasome inhibitors. (b) Effect of ABT-199–induced integrated stress response on NOXA expression.

AgentD consistently generates more detailed and mechanistically accurate responses. For example, when asked about the synergy mechanism between venetoclax and proteasome inhibitors, the RAG-augmented answer includes key elements such as ISR activation, ATF3/ATF4-mediated transcription of NOXA, and downstream effects involving BIM, BAX/BAK activation, and mitochondrial membrane permeabilization,closely aligning with the mechanistic explanation presented in the source paper (Figure 3a). In contrast, vanilla GPT-4o provides a more generic explanation, omitting critical components such as the ISR pathway and transcriptional regulators.

A similar pattern is observed when inquiring about how ABT-199 influences NOXA expression through ISR. AgentD correctly references eIF2α phosphorylation, TP53 independence, and the ATF3/ATF4 regulatory cascade. Although the GPT-4o response is fluent, it lacks these essential mechanistic details. In Figure 3, AgentD’s context-aware answers are highlighted in green, whereas GPT-4o’s generic responses appear in gray.

To quantitatively assess these observed differences, we measured the semantic similarity between responses generated with and without RAG and human-annotated reference answers derived from the literature using BERTScore.^54^ The reference answers were curated by the authors through careful reading of the relevant source papers.^53,55^ BERTScore quantifies contextual similarity by comparing the embeddings of model-generated and reference texts. We employed SciBERT^56^ as the embedding encoder because it is pretrained on a large corpus of scientific publications from Semantic Scholar, capturing richer representations of biomedical and technical terminology than general-domain encoders such as RoBERTa.^42^

As shown in Table 2, AgentD consistently outperforms the corresponding vanilla language models across both GPT-4o and DeepSeek-Chat backends. For GPT-4o, the use of RAG increases the mean F1-score from 0.63 ± 0.01 to 0.70 ± 0.05, primarily driven by a substantial gain in Precision (0.72 vs 0.61). This indicates that AgentD more effectively suppresses unsupported or generic statements during question answering. A similar trend is observed with DeepSeek-Chat: AgentD improves the F1-score from 0.67 ± 0.01 in the vanilla model to 0.70 ± 0.05, again with a noticeable increase in Precision (0.69 vs 0.65). These results show that integrating RAG and structured reasoning enables AgentD to produce more faithful and literature-grounded responses.

**Table 2:** Comparison of BERTScores between AgentD (with RAG) and Vanilla Language Models (without RAG)^a^

**Method**	**Precision**	**Recall**	**F1-score**
AgentD (GPT-4o, w/RAG)	0.719 ± 0.048	0.686 ± 0.064	0.701 ± 0.046
Vanilla GPT-4o (w/o RAG)	0.609 ± 0.018	0.661 ± 0.039	0.633 ± 0.024
AgentD (DeepSeek-Chat, w/RAG)	0.685 ± 0.064	0.717 ± 0.042	0.700 ± 0.051
Vanilla DeepSeek-Chat (w/o RAG)	0.645 ± 0.021	0.698 ± 0.020	0.670 ± 0.012

a Values are averaged across six question–answer pairs. Results for two representative pairs are shown in Figure 3, and additional examples are provided in Supplementary Information Section 1.

### Seed Molecule Generation

The initial library of molecules serves as a critical foundation for exploring chemical space and identifying candidates for further optimization. To construct this seed molecule pool, AgentD leverages two complementary generation strategies. REINVENT enables de novo molecule generation, allowing for broad and unbiased exploration of chemical space without the need for an input structure. Mol2Mol performs conditional generation, producing analogs that are structurally similar to a specified input molecule. In our workflow, the existing drug identified during the data extraction phase, such as venetoclax, is used as input for Mol2Mol, enabling the agent to focus on chemically relevant and biologically meaningful regions of the search space.

After retrieving the SMILES of the known drug, AgentD automatically integrates it into a configuration file and executes both REINVENT and Mol2Mol to generate the initial seed molecules. As shown in Figure 4, REINVENT-generated molecules are widely distributed across the chemical space, reflecting its exploratory capabilities, whereas Mol2Mol-generated molecules are more tightly clustered around the input molecule, enabling targeted exploration near known active scaffolds. This seed library serves as the starting point for downstream tasks such as property prediction, refinement, and structure generation.

**Figure 4:**
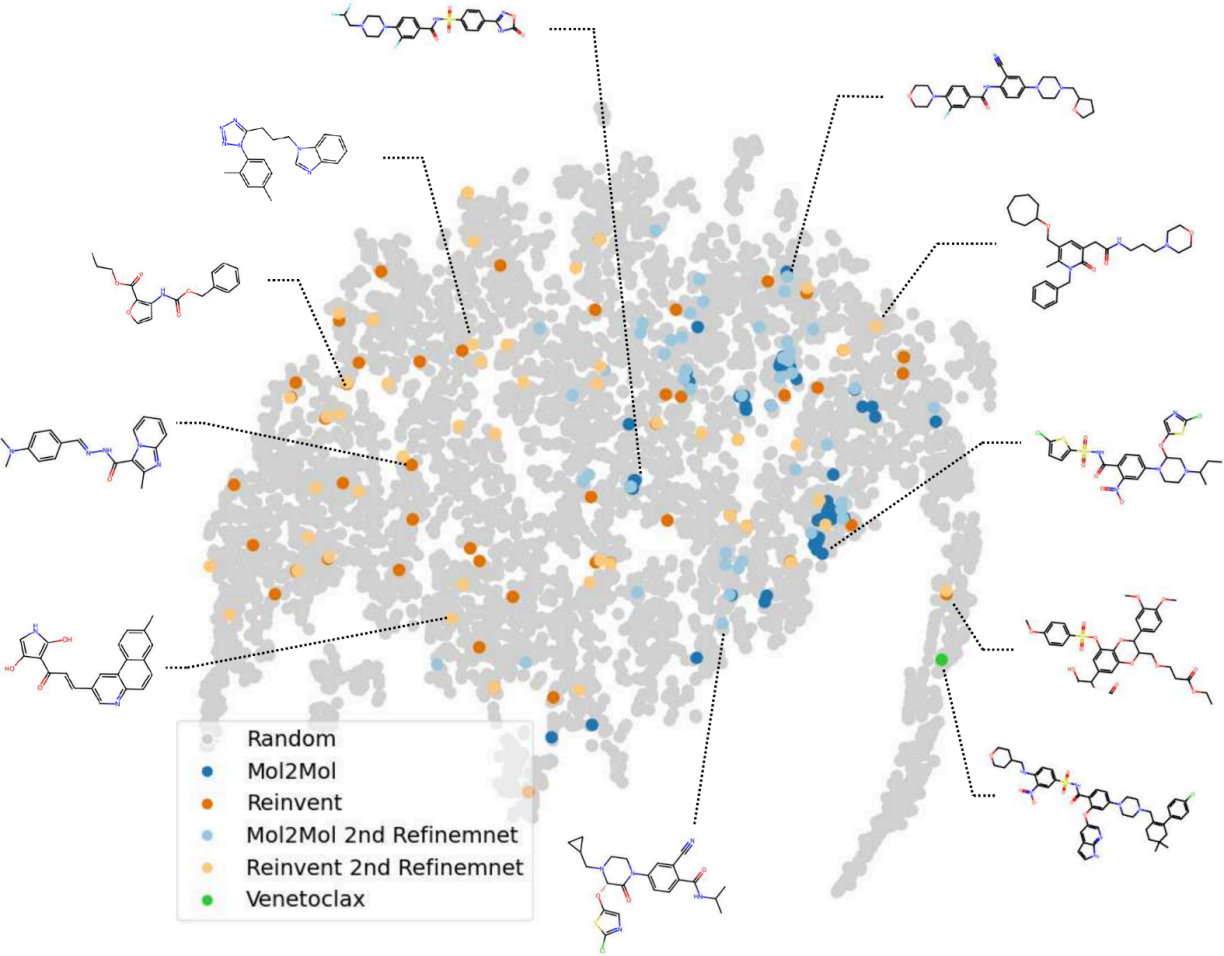
t-SNE visualization of chemical space, based on Mordred-derived molecular descriptors,^57^ including seed molecules generated by AgentD (Mol2Mol, REINVENT), refined molecules after property-aware optimization, 5,000 randomly sampled compounds from ChEMBL, and the reference drug venetoclax.

### Property-Aware Molecular Refinement

To enhance the quality of the seed molecule pool, AgentD performs property-driven refinement of SMILES structures by identifying and addressing limitations that compromise their drug-likeness. Using the Deep-PK model API,^48^ the agent predicts 67 ADMET properties, as well as 7 general physicochemical properties. A complete list of predicted ADMET properties is provided in Supplementary Table S2. In parallel, AgentD uses the BAPULM model^49^ to estimate binding affinity (p*K*_d_).

Based on these predictions, AgentD identifies unfavorable molecular properties relevant to drug development, such as low permeability or high toxicity, and proposes targeted structural edits to improve them. A comprehensive list of weakness-associated properties is provided in Supplementary Table S3. We demonstrate this refinement process over two iterations, beginning with an initial set of 100 molecules. These iterations produced 99 and 95 additional valid SMILES, respectively, which were added to the candidate pool. For instance, a molecule is initially flagged for poor permeability due to a low predicted log *P*_app_ value (Figure 5a). In response, AgentD suggested replacing a hydroxyl group with a methyl group in the first round, followed by substituting a sulfonamide with an amine in the second - both changes contributed to improved predicted permeability. Figure 5d illustrates another case of successful refinement, where the targeted weak properties, such as toxicity and permeability, were improved at each step. If resources permit, further iterations can be used to improve weak properties and strengthen the molecule’s potential as a drug candidate.

**Figure 5:**
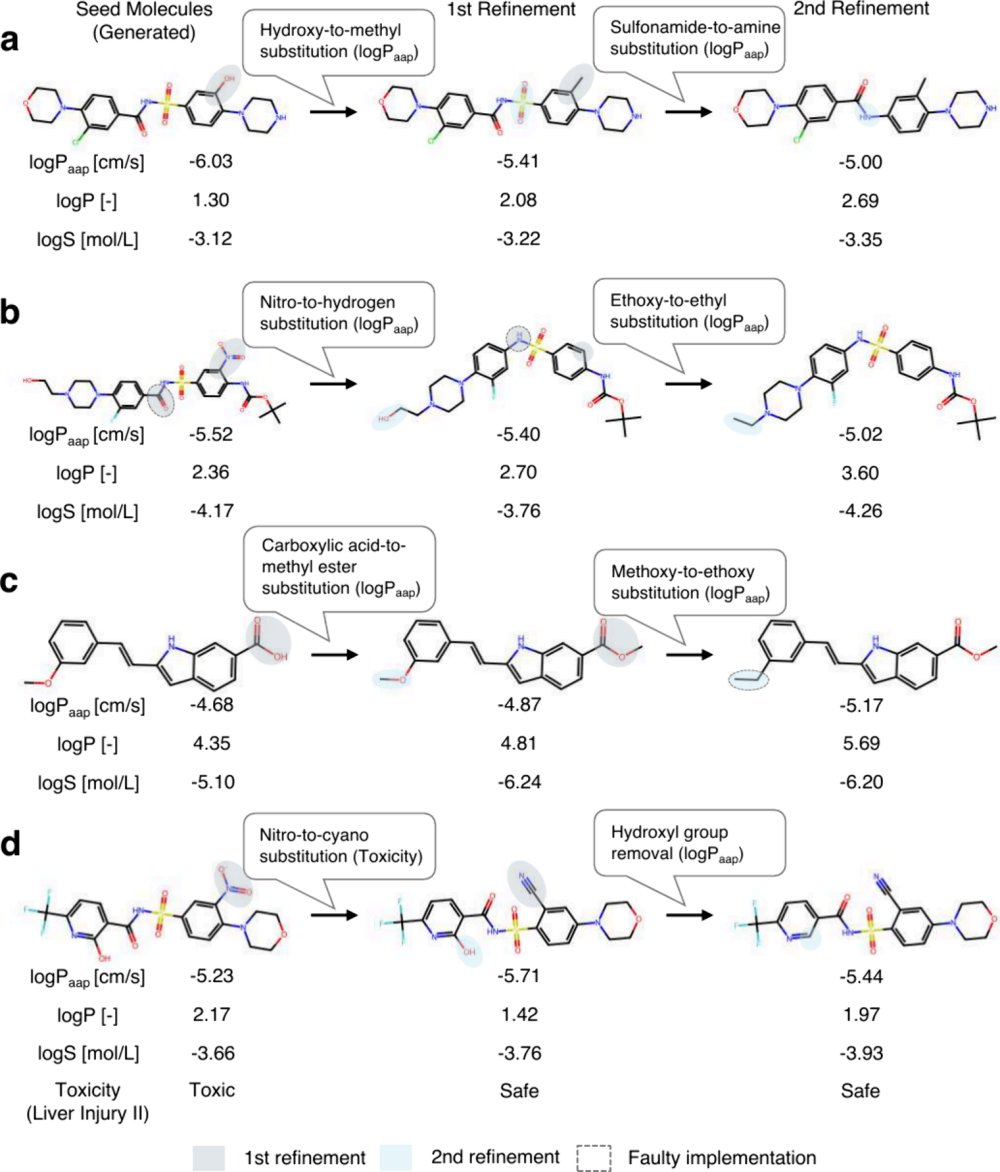
Property-aware molecular refinements by AgentD. (a) Iterative changes improve permeability. (b) An unintended modification leads to improved permeability. (c) An intended modification fails to improve permeability. (d) Iterative changes improve both toxicity and permeability.

Refinements are not always successful or implemented as intended. Two common failure modes are observed: (i) unintended SMILES modifications that diverge from the agent’s stated intent, and (ii) correctly executed modifications that fail to improve the target property. For case (i), in Figure 5b, the agent intended a nitro-to-hydrogen substitution and successfully applied it, but also unintentionally removed a carbonyl group; nonetheless, the predicted permeability improved. Additionally, in Figure 5c, an intended methoxy-to-ethoxy substitution was improperly implemented, resulting in the loss of an oxygen atom and decreased permeability. For case (ii), even when the modification is applied correctly, the desired outcome may not be achieved, as seen in the first-round refinement of Figure 5c, where the permeability worsened despite the intended structural change.

In the first refinement round, 57 out of the initial 100 molecules were identified as having low permeability. Among these, approximately 44% showed improved log *P*_app_ values after refinement, 26% declined, and 30% remained unchanged (Figure 6a). In the second round, 52% improved, 17% declined, and 31% were unchanged. For the molecules that showed target property improvement, the median log *P*_app_ increased from −5.33 to −5.03 (Figure 6b). Although the magnitude of change is modest, the upward trend is clearly visible within just two iterations. Toxicity improvements were relatively more limited: 24% and 20% of high-toxicity molecules showed improvement in the first and second rounds, respectively, with most remaining toxic despite modification (Figure 6c).

**Figure 6:**
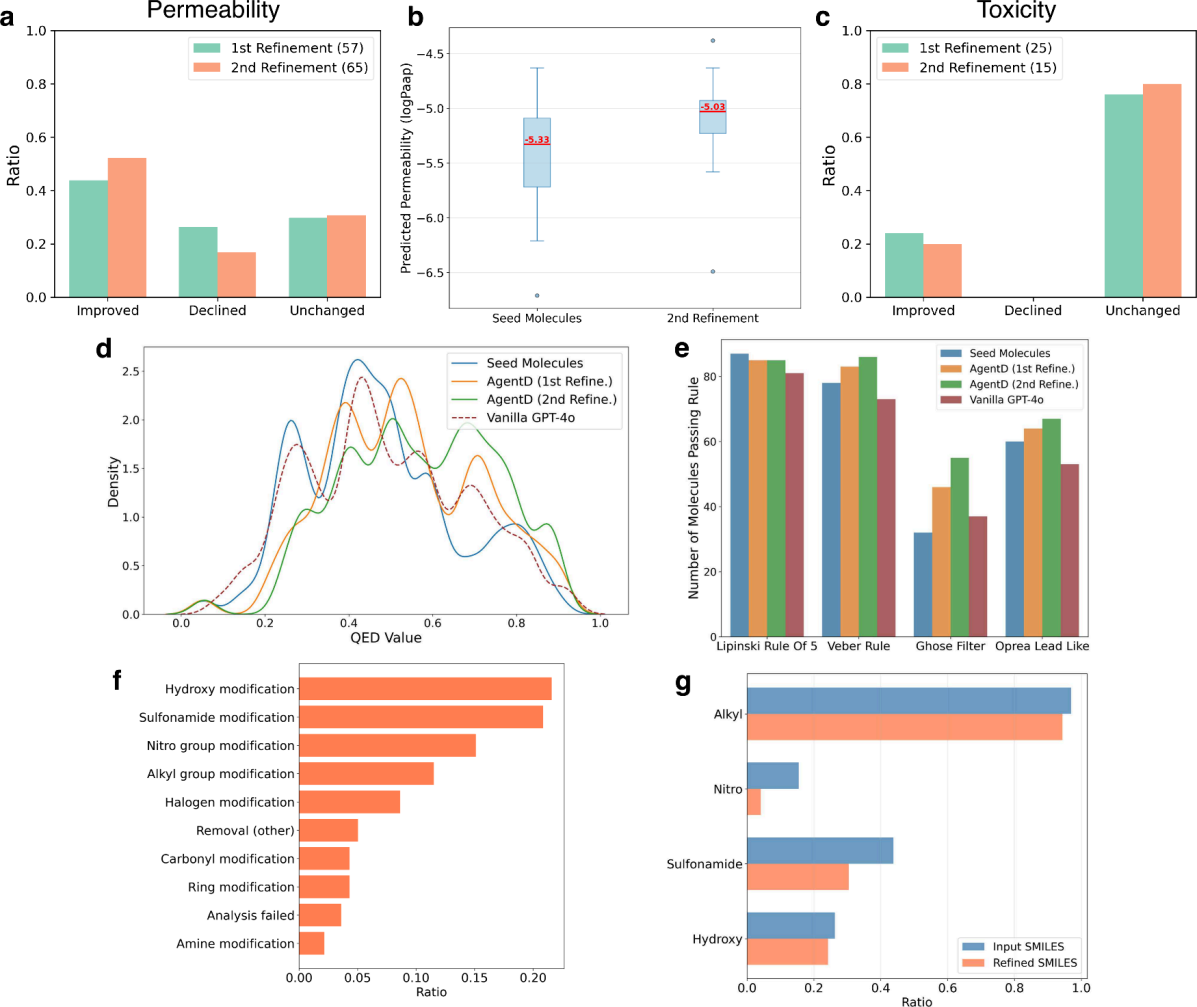
Property-aware molecular refinement results. (a) Proportion of molecules with improved, declined, or unchanged permeability (logP_app_). (b) Boxplot of logP_app_ for entries showing improvement. (c) Molecular refinement patterns for molecules with improved permeability. (d) QED distribution shifts across iterations (vanilla GPT-4o indicates refinement solely through direct queries to GPT-4o). (e) Counts of molecules satisfying empirical drug-likeness rules (definitions in Methods). (f) Fraction of molecules modified for permeability; “Analysis failed” indicates unresolved substructure differences by the substructure algorithm. (g) Functional-group frequencies in input vs refined molecules across iterations (input = input for refinement; refined = postrefinement outputs).

Despite occasional local failures, as illustrated in Figure 5b and c, the overall impact of refinement is positive across the molecule pool. This is supported by increased QED (Quantitative Estimate of Drug-likeness) scores, which reflect overall drug-likeness based on empirical physicochemical properties.^58^ As shown in Figure 6d, the distribution of high-QED molecules expanded with each refinement iteration: the number of molecules with QED > 0.6 increased from 34 in the original set to 49 after the first update, and to 55 after the second.

As shown in Figure 6e, LLM-based refinement increased the proportion of molecules meeting empirical drug-likeness and lead-likeness criteria, including Lipinski’s Rule of Five, Veber’s rule, Ghose’s filter, and Oprea’s lead-likeness filter.^59–62^ These rules assess different aspects of molecular suitability: Lipinski’s and Veber’s rules emphasize oral bioavailability and permeability, Ghose’s filter captures physicochemical ranges observed in marketed drugs, and Oprea’s filter identifies compounds suitable as lead-like starting points for optimization. After refinement, more molecules satisfied Veber’s rule (78 to 86) and Oprea’s filter (60 to 67). The most notable improvement was observed for Ghose’s filter, with the number of passing molecules increasing from 32 to 55 (∼72% increase). These results indicate that refinement helped expand the chemical space toward drug-like and lead-like regions, particularly in terms of Ghose’s statistical descriptors. Compliance with Lipinski’s rule remained largely unchanged; this outcome is further discussed in the Discussion section.

AgentD tends to prioritize edits to polar or electron-withdrawing groups, such as hydroxyls, sulfonamides, and nitro groups, when attempting to improve permeability-related properties. Figure 6f shows the distribution of SMILES modifications for entries with permeability as the target weakness property. The most frequent edits involved hydroxyl groups (30 cases, 15.5%), followed by sulfonamide substitutions (29 cases, 15.0%) and nitro group changes (21 cases, 10.8%). Other commonly modified motifs included alkyl groups (8.3%) and halogens (6.2%), and together these five categories account for 55.7% of all refinements. Also, AgentD’s refinement behavior does not merely reflect functional-group prevalence. For instance, despite nitro groups being less common than alkyl, sulfonamide, or hydroxyl groups, nitro edits still represent a substantial portion of modifications (10.8%; Figure 6g). This targeted preference for polar and electron-withdrawing group edits contrasts sharply with the behavior of a vanilla GPT-4o model, which predominantly applies halogen and alkyl substitutions and does not exhibit polar functional-group enrichment in the absence of property-aware guidance (see Supplementary Figure S9).

### 3D Structure Generation

After refining the seed molecule pool through property-aware modifications, we applied empirical filtering criteria to select candidates for 3D structural evaluation. The purpose of this filtering scheme is not to identify a final drug candidate, but to illustrate the agent’s capability to perform key tasks in the drug discovery pipeline. In our workflow, molecules are shortlisted if they (i) satisfy Oprea’s lead-likeness filter, (ii) pass at least two of the three drug-likeness rules (Lipinski’s Rule of Five, Veber’s rule, and Ghose’s filter), (iii) achieve a QED score above 0.55, and (iv) have a predicted p*K*_d_ value greater than 6.0.

The molecule shown in Figure 7 satisfies the lead-likeness requirement, passes all three drug-likeness rules, has a QED score of 0.68, and achieves a predicted p*K*_d_ of 6.18. This is simply a representative candidate for further analysis. It is important to note that these empirical rules may not always align, and molecules rarely satisfy all of them simultaneously. In fact, only one molecule in our set passed all three rules, which we selected as an illustrative example for 3D visualization. Additional examples are provided in Supplementary Figure S7.

**Figure 7:**
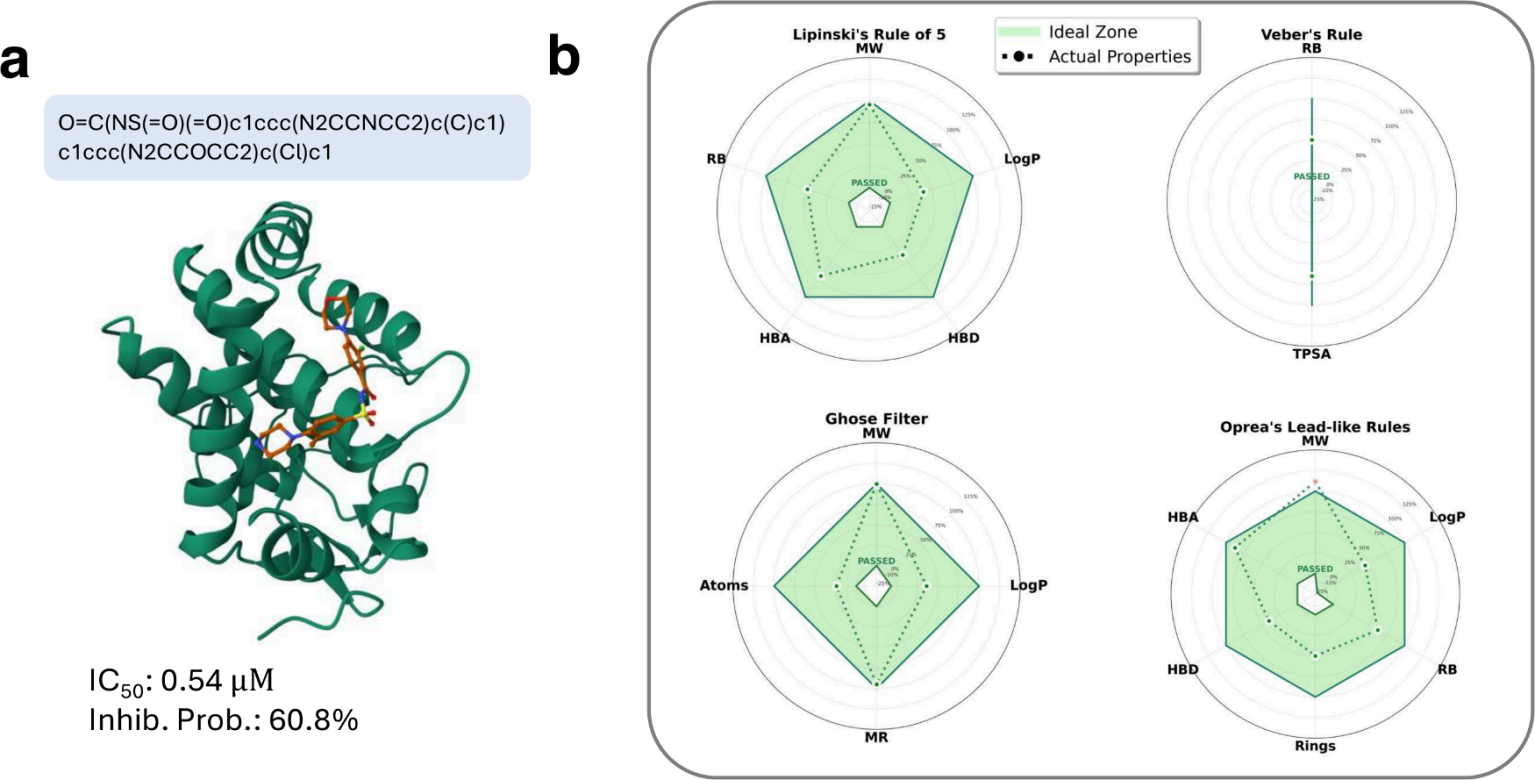
Protein–ligand structure generation and evaluation. (a) A representative 3D complex predicted by Boltz-2, with estimated IC_50_ and inhibitor probability. (b) Radar plot showing empirical rule-based evaluation. Green dotted lines indicate the thresholds for each rule, while the purple solid line represents the properties of the selected molecule. See Methods and Table S4 for criteria.

AgentD uses Boltz-2 as a tool to generate 3D protein–ligand complex structures using the ligand’s SMILES and the target protein’s FASTA sequence. These structures provide a foundation for more rigorous downstream evaluations, such as molecular docking, MD simulations, and free energy perturbation analyses, to assess binding stability, conformational flexibility, and interaction specificity under biologically relevant conditions. In addition to 3D structure, Boltz-2 returns estimated binding metrics, including logIC_50_ values and inhibitor probabilities. In Figure 7, the predicted IC_50_ (0.54 μM) and inhibitor probability indicate good submicromolar binding affinity, making the molecule a suitable candidate for further validation using molecular dynamics (MD) simulations.

### Comparison Across Language Models

AgentD can operate with a variety of LLMs, including those from OpenAI, Anthropic, and DeepSeek. In practice, the framework is optimized for GPT-4o. We tested AgentD using GPT-4o, Claude-3.7-Sonnet, and DeepSeek-Chat.^63–66^ DeepSeek-Chat, an open-source model, successfully executed all six tasks and demonstrated behavior broadly comparable to GPT-4o. In contrast, Claude-3.7-Sonnet exhibited failure modes in two tasks: domain-specific question answering and molecular refinement.

In the question-answering task, Claude-3.7-Sonnet encountered syntax errors when parsing PDF files. AgentD, powered by GPT-4o, achieved higher BERTScores than AgentD with DeepSeek-Chat (Table 2). Among the vanilla language models, DeepSeek-Chat outperformed GPT-4o, highlighting that the performance gain provided by AgentD is more pronounced when using GPT-4o.

In the molecular refinement task, AgentD with Claude-3.7-Sonnet occasionally became trapped in repeated attempts to generate valid updated SMILES, despite explicit instructions to avoid infinite retries (see Supplementary Figure S4). For the quantitative comparison of property-aware molecular refinement shown in Figure 8, we report results from a verified successful run of Claude-3.7-Sonnet to ensure a fair comparison.

**Figure 8:**
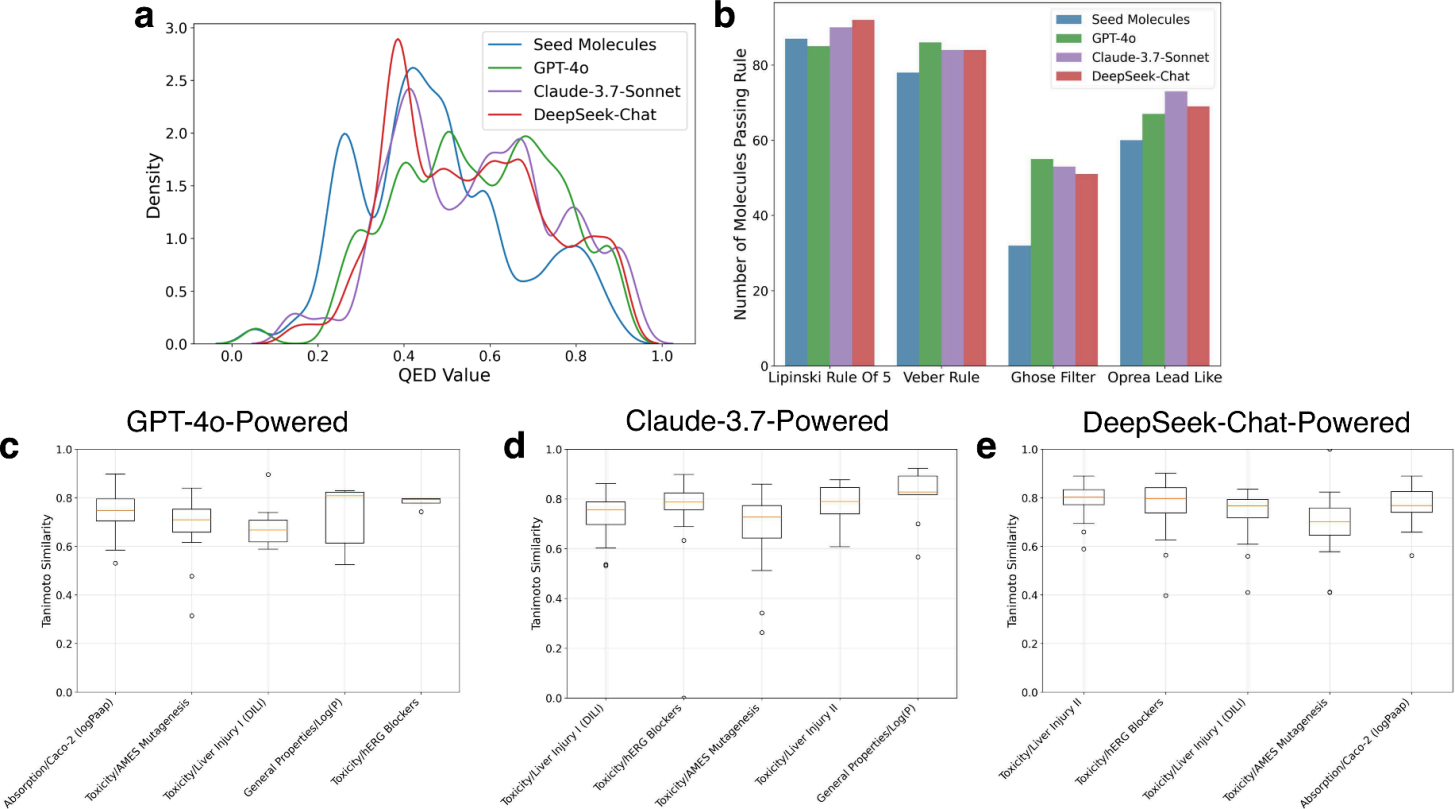
Comparison of AgentD performance across different language models. (a) Distribution of QED values before and after two iterations of property-aware molecular refinement for AgentD powered by GPT-4o, Claude-3.7-Sonnet, and DeepSeek-Chat. (b) Histogram showing the number of molecules passing empirical lead- and drug-like rules. (c–e) Tanimoto similarity between the original and updated SMILES for the top five most frequently targeted weakness properties for AgentD powered by GPT-4o (c), Claude-3.7-Sonnet (d), and DeepSeek-Chat (e).

All three model cases improved the QED of seed molecules after two iterations of property-aware refinement (Figure 8a). Starting from an average QED of 0.47, AgentD, powered by GPT-4o, increased the mean value to 0.57, Claude-3.7-Sonnet increased it to 0.59, and DeepSeek-Chat increased it to 0.55. While all models demonstrated meaningful improvements, DeepSeek-Chat achieved a slightly smaller gain relative to the others. The seed set contained 23.0% of molecules with QED > 0.6, which increased to 45.7% with GPT-4o, 44.9% with Claude-3.7-Sonnet, and 40.0% with DeepSeek-Chat after the second refinement step.

The rule-based evaluation shows a similar trend across the three LLMs, with the most evident increase observed in the number of molecules passing the Ghose filter (Figure 8b). Claude-3.7-Sonnet and DeepSeek-Chat do not exhibit the slight deterioration in Lipinski’s Rule of Five observed with GPT-4o, although both models yield slightly fewer molecules passing Veber’s rule.

Each language model exhibits some variability in the selection of “weakness” properties during molecular refinement. Figures 8c–e report the Tanimoto similarity^67,68^ between the original SMILES and the updated SMILES for the top five most frequently targeted weakness properties. For AgentD powered by GPT-4o, the top five properties were logP_app_, AMES mutagenicity, liver injury I toxicity, logP, and hERG blocker toxicity, with median similarities of 0.71, 0.71, 0.67, 0.81, and 0.79, respectively. Despite model-specific differences, the selected properties largely arise from a consistent subset of the 75 property end points, indicating broadly aligned refinement priorities. AgentD with Claude-3.7-Sonnet prioritized liver injury I toxicity, hERG blocker toxicity, AMES mutagenicity, liver injury II toxicity, and logP, with median similarities of 0.76, 0.79, 0.73, 0.79, and 0.83. For AgentD with DeepSeek-Chat, the top five properties were liver injury II toxicity, hERG blocker toxicity, liver injury I toxicity, AMES mutagenicity, and logP_app_, with median similarities of 0.80, 0.80, 0.77, 0.70, and 0.77. Although all three models generally target similar toxicity- and permeability-related weaknesses, the specific ranking of properties and the degree of structural change.

## Discussion

### Property Awareness Improves Drug-Likeness

Weakness-property awareness substantially improves drug-likeness, even though drug-likeness is not explicitly used as an optimization target. Refinement via a vanilla LLM, without property-aware guidance, yields little measurable improvement (Figure 6d–e). In contrast, incorporating weakness-property information leads to pronounced gains, indicating that providing explicit context about what is deficient in a molecule enables more targeted and pharmacologically meaningful edits. This functions similarly to chain-of-thought reasoning: giving the LLM stepwise situational cues (e.g., “this is the weakness, so modify accordingly”) helps steer its structural decisions in a more directed and interpretable manner.

AgentD’s refinement pattern also does not simply mirror the prevalence of functional groups in the data set (Figure 6f–g). Instead, the model displays distinct preferences in the types of chemical transformations it applies, suggesting that the observed patterns arise from its learned reasoning rather than from data set-driven frequencies.

The benefit of property-aware refinement is consistent across all LLMs used as the agent’s core engine (Figure 8a–b). Whether using GPT-4o, Claude-3.7-Sonnet, or DeepSeek-Chat, the refinement strategy reliably increases drug-likeness. This indicates that the performance gains stem from the refinement paradigm itself rather than from model-specific idiosyncrasies.

### Challenges in Molecular Refinement

The property-aware molecular refinement module also shows limitations. Molecular optimization remains intrinsically complex due to interdependencies among properties. For example, in Figure 5a and b, predicted apparent permeability (logP_app_) is influenced by lipophilicity (logP), consistent with Overton’s rule,^69^ whereas aqueous solubility (logS) does not follow a consistent trend and may either increase or decrease. These observations highlight the complex, nonlinear interplay among lipophilicity, solubility, and permeability. Similarly, the first-round refinement in Figure 5d mitigates predicted liver toxicity but worsens permeability. These trade-offs highlight the fundamental challenge of multiproperty optimization in drug design. These findings highlight the importance of incorporating multiobjective optimization in future iterations to better balance competing pharmacological goals. Despite these complexities, AgentD achieves a net improvement in overall drug-likeness across the molecule pool, as shown in Figure 6c and d.

Molecular optimization is also target-dependent, as multiple interacting physicochemical properties influence outcomes. Across the three therapeutic targets, BCL-2, JAK-2, and thrombin, drug-likeness improves overall, as reflected by QED distributions and rule-based filters, though the improvement patterns differ. For BCL-2 and thrombin, the largest gains occur under the Ghose filter, while Lipinski’s Rule of Five shows only minor improvement, likely because oral bioavailability was rarely selected as a weakness (Supplementary Table S3). By contrast, JAK-2 shows stronger gains under Lipinski’s criteria but minimal change under the Ghose filter. These differences highlight the intrinsic complexity of molecular refinement, where multiple interdependent properties are convoluted.

### Effective Retrieval and Generation

The agent reliably retrieves protein and ligand data from both structured databases and web sources. While our current implementation focuses on targeted extraction, future extensions could incorporate large-scale literature mining and relevance scoring to support broader knowledge synthesis.

For domain-specific question answering, AgentD’s RAG-augmented responses consistently outperform standard LLM outputs in both contextual accuracy and mechanistic completeness (Table 2). Because RAG performance depends strongly on the quality of retrieved literature, expanding retrieval beyond open-access sources may further enhance results. Our current implementation uses a deliberately minimal retrieval pipeline—without filters on publication date, venue quality, or citation metrics—yet still provides clear gains in factual grounding and mechanistic fidelity. These findings demonstrate that even a raw, loosely constrained RAG setup can substantially improve scientific question answering. We further evaluated alternative retrieval strategies, including citation-ranked and publication-year-filtered approaches (Supplementary Table S2), which also outperform the vanilla LLM baseline without RAG.

For molecule generation, AgentD integrates external models such as REINVENT and Mol2Mol using natural language-driven configuration updates, enabling seamless integration of new tools with minimal customization. By leveraging a language model to interpret and modify configuration files based on high-level instructions, the system can flexibly switch between different generative models or sampling strategies without manual code changes. This approach removes the need for model-specific hardcoded logic and allows for rapid adaptation to evolving architectures and file structures. The modular design of AgentD further ensures its long-term adaptability as generative modeling techniques continue to advance.

The structure generation task complements the pooling and refinement process by enabling 3D protein–ligand complex generation, which provides critical input for structure-based studies. Boltz-2 captures meaningful trends in ligand binding behavior, but its IC_50_ and inhibitor probability predictions should be interpreted as rough approximations that are useful for initial prioritization rather than substitutes for MD-based physical validation.

### Limitations and Future Directions

Despite its modular design and encouraging end-to-end performance, AgentD has several limitations. The workflow depends on external predictive tools such as Deep-PK, BAPULM, and Boltz-2, each with its own error profile and inherent uncertainties. Errors in these modules can propagate downstream, affecting both refinement decisions and compound prioritization. For instance, an inaccurate ADMET property prediction can lead to incorrect weakness determination and suboptimal molecular refinements. To mitigate this, we analyze outcomes at the group level rather than on a per-run or per-molecule basis. Despite these uncertainties, the overall drug-likeness, as measured by QED and empirical rules, shows clear improvement after iterative refinement. AgentD’s current goal is to generate a promising compound pool for further evaluation, making this approach suitable for its purpose. To enhance individual success rates, future iterations of AgentD should integrate more rigorous predictive tools as they become available. This rationale also underpins the framework’s modular design, allowing updated tools to be incorporated easily. In particular, predicted protein–ligand complex structures and affinity should be interpreted as screening-level signals and require downstream MD/free-energy validation prior to mechanistic or potency claims.

## Conclusion

In this study, we introduced AgentD, a modular, LLM-powered agent framework for automating and streamlining key tasks of the drug discovery pipeline. By integrating language model reasoning with domain-specific tools and databases, AgentD can: (i) retrieve relevant protein and compound data from structured databases and web sources; (ii) answer domain-specific scientific questions grounded in literature; (iii) generate diverse, context-aware molecules using both de novo and conditional models; (iv) predict pharmacologically relevant properties; (v) refine molecular representations through iterative, property-aware optimization; and (vi) construct protein–ligand complex structures for downstream simulations.

AgentD marks a step toward general-purpose, AI-driven scientific agents for therapeutic discovery. Its modular architecture supports seamless integration of new models and tools, ensuring continued adaptability as technologies evolve. As a necessary downstream validation step, MD simulations should be applied to physically validate AgentD-generated 3D complexes, ideally complemented by free-energy calculations when feasible. Future work will focus on automating this validation within the agent loop, along with multiobjective molecular optimization and conditional generation toward desired pharmacological profiles.

## Methods

### Large Language Model Agent

Our framework primarily employs OpenAI’s GPT-4o model^63^ as the main language model for all AgentD modules. GPT-4o is an optimized version of GPT-4, part of the Generative Pretrained Transformer family.^70^ In addition, we integrated Anthropic’s Claude-3–7-sonnet-20250219 and DeepSeek-Chat models. All models are accessed via their respective APIs and loaded dynamically using the LangChain library,^71^ which simplifies prompt management, response processing, and integration with external tools. All models are used with default settings provided by the API. For all API calls, no local GPU resources are required; however, for tool-specific tasks such as binding affinity prediction, SMILES generation, and protein–ligand structure generation, computations are performed on a single NVIDIA RTX 2080 Ti GPU.

### Database

We utilize two established bioinformatics resources, UniProt and ChEMBL, to access protein sequence data and small molecule representations, respectively. UniProt provides high-quality annotated protein sequences between species, enabling access to functionally characterized targets of therapeutic relevance.^43^ For each therapeutically relevant protein target (e.g., *EGFR* or *TP53*), the agent queries UniProt’s REST API, restricted to the *Homo sapiens* taxonomy (organism ID: 9606) to retrieve the corresponding amino acid sequence in FASTA format. When existing drugs are available for the target, we subsequently query ChEMBL to obtain SMILES representations of these known compounds.^44^ This ChEMBL query step is only executed if existing drugs for the target are identified through prior web searches; otherwise, this step is skipped.

### Retrieval-Augmented Generation

AgentD incorporates a lightweight Retrieval-Augmented Generation (RAG) workflow to provide literature-grounded responses during domain-specific question answering. In its current implementation, literature retrieval is intentionally kept minimalistic: the agent generates search keywords, and papers are retrieved solely through the Serper API using these keywords, without applying explicit filters on publication year, venue quality, or citation count. As a result, the retrieved documents may vary in quality and relevance.

Once retrieved, PDFs are processed with a CharacterTextSplitter (chunk size 1000 characters, 50-character overlap) to maintain contextual continuity across segments. Each chunk is embedded using EMBEDDING_MODEL and stored in a FAISS vector index to allow uniform and efficient retrieval. During the question-answering phase, the LLM performs similarity search over the FAISS index to identify the most relevant text segments, which are then supplied as grounding context for response generation.

### ADMET Properties

ADMET properties encompass Absorption, Distribution, Metabolism, Excretion, and Toxicity characteristics that are critical for drug development.^72,73^ These pharmacologically relevant descriptors determine how a compound is absorbed into the bloodstream, distributed across tissues, metabolized by enzymatic systems, eliminated from the body, and whether it poses potential toxic effects. Early prediction of ADMET properties is essential for prioritizing compounds with favorable biopharmaceutical and safety profiles, thereby reducing downstream attrition during drug development.^74,75^

As part of this multistage assessment, we integrated Deep-PK, an ADMET prediction framework that operates on SMILES input and internally employs a Message Passing Neural Network (MPNN) to capture the atomic and topological features of each molecule.^48^ Candidate ligands, whether recovered from ChEMBL or generated, are submitted to the Deep-PK REST API via their SMILES strings, and the resulting ADMET profiles are parsed to prioritize compounds with favorable pharmacokinetic and safety attributes. The complete set of predicted properties is summarized in Supplementary Table S2.

A tolerance mechanism was implemented for Deep-PK API requests. After submitting SMILES, the system polls for job completion at fixed intervals (30 s) up to a maximum wait time (500 s). API errors or missing job IDs are logged and returned without interrupting the pipeline, and running jobs are retried until completion or timeout.

### Binding Affinity

Binding affinity to the biological target is a fundamental determinant of therapeutic potential, serving as a key predictor of drug potency and selectivity. We employ two complementary strategies for affinity prediction. In the property prediction task, the sequence-based BAPULM model employs a dual encoder architecture to estimate the dissociation constant (*K*_d_) from protein amino acid sequences and ligand SMILES representations. It integrates two domain-specific pretrained language models: ProtT5-XL-U50 for proteins and MolFormer for small molecules.^76,77^ Each encoder generates latent embeddings tailored to its respective input, which are then projected into a shared latent space using learnable feedforward projection heads. Subsequently, a predictive head processes these joint representations to estimate the binding affinity, reported as p*K*_d_ = – log_10_(*K*_d_).^49^ Given its computational efficiency and reliance solely on sequence-level inputs, it is well-suited for early stage screening of large ligand libraries based on predicted binding affinity.

In the structure generation task, the structure-based model Boltz-2 begins with the same sequence and SMILES input but internally generates 3D protein–ligand complex structures. These conformations are then used to predict the half-maximum inhibitory concentration (IC_50_), reflecting the inhibitory efficacy of a compound in biochemical assays.^78^ In addition to regression-based affinity values, Boltz also outputs inhibitor probability scores, indicating the likelihood that a given ligand acts as an active binder. Although *K*_d_ and IC_50_ originate from different experimental setups, they are related through the Cheng–Prusoff equation^79^ (eq 1).

$$K_{i}=\frac{{\mathrm{IC}}_{50}}{1+\frac{\left[S\right]}{K_{m}}}$$ 1

where *K*_*i*_ approximates *K*_d_ under certain biochemical conditions.

### SMILES Generation

We utilize two molecular generators: REINVENT for de novo molecular design and Mol2Mol for molecular optimization.^45–47^ Both generators utilize recurrent neural networks and transformer architectures and are embedded within machine learning optimization algorithms, including reinforcement learning and transfer learning.^46,80^ Both models were implemented using the REINVENT4 package and configured to generate one molecule per input SMILES (‘num_smiles = 157’), while retaining only unique molecules (‘unique_molecules = true’) with canonicalized SMILES to eliminate duplicates.

REINVENT performs de novo molecular generation using sequence-based models that capture SMILES token probabilities in an autoregressive manner. The models are trained via teacher-forcing on large SMILES data sets to learn chemical syntax and generate valid molecules.^81^

Mol2Mol performs conditional generation, accepting input SMILES strings and generating structurally similar molecules within user-defined similarity constraints.^47^ The transformer-based model was trained on over 200 billion molecular pairs from PubChem with Tanimoto similarity ≥ 0.50. Training employed ranking loss to directly link negative log-likelihood to molecular similarity. The model supports multinomial sampling with temperature control (set to 1.0 in this study).

### Protein–Ligand Complex Structure

Boltz-2 is a structure-based deep learning model which can jointly predict 3D protein–ligand complex structures by integrating protein folding and ligand binding into a unified framework.^51^ The model takes as input a protein FASTA sequence and a ligand SMILES string, and simultaneously infers the full atomic conformation of the protein as well as the bound pose of the ligand within the predicted binding pocket. Unlike traditional docking pipelines that require experimentally resolved protein structures, Boltz-2 performs *ab initio* structure prediction, enabling end-to-end modeling from sequence alone.

Its architecture builds upon the Evoformer^20^ stack and SE(3)-equivariant transformer modules, incorporating interleaved attention mechanisms to capture long-range dependencies both within the protein sequence and between the protein and ligand.^50,51,82^ This allows Boltz-2 to reason over flexible ligand conformations and protein-side chain rearrangements in a physics-aware manner. By directly predicting all-atom 3D coordinates, the model enables rapid generation of realistic protein–ligand complexes suitable for downstream scoring.

### Evaluation Metrics

To evaluate the drug-likeness, lead-likeness, and pharmacokinetic relevance of the generated molecules, we applied four widely used rule-based filters: Lipinski’s Rule of Five,^59^ Veber’s Rule,^60^ the Ghose filter,^61^ and Oprea’s Lead-like Rule.^62^ The first three rules assess general drug-likeness, while Oprea’s rule specifically addresses lead-likeness as an early stage starting point for optimization. The detailed numerical thresholds for each rule are summarized in Table S4 of the Supporting Information. All descriptors were computed using RDKit.

Lipinski’s Rule of Five includes five criteria: molecular weight (MW) ≤ 500 Da, LogP ≤ 5, hydrogen bond donors (HBD) ≤ 5, hydrogen bond acceptors (HBA) ≤ 10, and rotatable bonds ≤ 10. A molecule was considered compliant if it satisfied at least four of the five conditions. Although the original formulation included only the first four parameters, the rotatable bond threshold has since been adopted to better capture oral absorption potential.

Veber’s rule assesses polarity and flexibility using two thresholds: topological polar surface area (TPSA) ≤ 140 Å^2^ and rotatable bonds ≤ 10. Both conditions must be met for compliance.

The Ghose filter identifies drug-like chemical space based on statistical distributions observed in marketed drugs. Its thresholds include MW between 160–480 Da, LogP between −0.4 and 5.6, molar refractivity (MR) between 40–130, and heavy atom count between 20–70. Molecules meeting these ranges are considered to occupy favorable physicochemical space for drug development.

Oprea’s Lead-like Rule defines chemical properties suitable for “lead” compounds—molecules that may not yet meet full drug-likeness but serve as starting points for further optimization. Criteria include MW between 200–450 Da, LogP between −1 and 4.5, HBD ≤ 5, HBA ≤ 8, rotatable bonds ≤ 8, and aromatic rings ≤ 4, with allowance for one violation. Compared to drug-like rules, these thresholds are narrower, reflecting the preference for smaller, less lipophilic molecules that can be synthetically elaborated into drug candidates.

## Technology Use Disclosure

We used ChatGPT and Claude to help with grammar and typographical corrections during the preparation of this preprint manuscript. The authors have carefully reviewed, verified, and approved all content to ensure accuracy and integrity.

## Supplementary Material



## Data Availability

The code that supports this study’s findings can be found in the following publicly available GitHub repository: https://github.com/hoon-ock/AgentD.

## Notes

Supporting Information is available and includes the following sections: Domain-specific Question Answering, Failure Cases with Claude-3.7-Sonnet, ADMET Properties, Identified Unfavorable Drug Properties, JAK-2 for Myelofibrosis, Thrombin for Thrombosis, Empirical Filters, Structure Generation Examples, LLM-based Molecule Optimization Approach, Uncertainty in Prediction Tools, Baseline Structural Modification, and Strategy–Structure Alignment Analysis. (PDF)
