# Supplementary material for: Large Language Model Agent for Modular Task Execution in Drug Discovery
Source: J Chem Inf Model. 2026 Feb 9;66(4):2055–68. doi: 10.1021/acs.jcim.5c02454 (PMC12933718; doi:10.1021/acs.jcim.5c02454)
Supplement: Supplementary file 1 [file ci5c02454_si_001.pdf]

Supporting Information: Large Language Model Agent for Modular Task Execution in Drug Discovery

Janghoon Ock,[†,‡,⊥] Radheesh Sharma Meda,[†,⊥] Srivathsan Badrinarayanan,[†] Neha S Aluru,[¶] Achuth Chandrasekhar,[§] and Amir Barati Farimani[*,‖]

†*Department of Chemical Engineering, Carnegie Mellon University, Pittsburgh, PA, USA*

‡*Department of Chemical and Biomolecular Engineering, University of Nebraska–Lincoln, Lincoln, NE 68588, USA*

¶*School of Engineering Medicine, Texas A&M University, Houston, TX, USA*

§*Department of Material Science and Engineering, Carnegie Mellon University, Pittsburgh, PA, USA*

‖*Department of Mechanical Engineering, Carnegie Mellon University, Pittsburgh, PA, USA*

⊥*These authors contributed equally to this work.*

E-mail: barati@cmu.edu

Contents

S1 Domain-specific Question Answering **S2**

S2 Failure Cases with Claude-3.7-Sonnet **S9**

S3 ADMET Properties **S10**

S4 Identified Unfavorable Drug Properties **S15**

S5 JAK-2 for Myelofibrosis **S16**

S6 Thrombin for Thrombosis **S18**

S7 Empirical Filters **S20**

S8 Structure Generation Examples **S22**

S9 LLM-based Molecule Optimization Approaches **S24**

S10 Uncertainty in Prediction Tools **S26**

S11 Baseline Structural Modification **S28**

S12 Strategy–Structure Alignment Analysis **S29**

References **S31**

S1 Domain-specific Question Answering

We conducted an additional evaluation question that examined AgentD's ability to extrapolate mechanistic findings to broader therapeutic contexts by asking about the implications of combining ABT-199 and proteasome inhibitors for treating solid tumors compared to hematologic malignancies. This question tests the system's capacity to synthesize domain-specific knowledge and provide clinically relevant insights beyond the immediate scope of the reference paper. Figure S1 shows the actual responses from both AgentD with RAG and the standard GPT-4o model to illustrate these differences in clinical reasoning and mechanistic understanding.

We evaluated responses using the same Weller et al. study, which primarily focuses on the mechanistic basis of drug synergy through NOXA upregulation and MCL-1 inhibition.[S1] While this question extends beyond the paper's direct experimental scope, it remains

grounded in the mechanistic principles described in the work. Both AgentD with RAG and the standard GPT-4o model were asked to discuss the therapeutic implications for different cancer types.

The RAG-augmented response demonstrates superior clinical reasoning by directly connecting the paper's mechanistic findings to therapeutic applications. AgentD correctly identifies that the combination could "significantly impact the treatment of both solid tumors and hematologic malignancies" through the dual mechanism of direct BCL-2 inhibition and indirect MCL-1 inhibition via NOXA activation. Importantly, the response provides specific examples, noting effectiveness in "solid tumors, such as soft tissue sarcoma" and established efficacy in hematologic malignancies, including "chronic lymphocytic leukemia and multiple myeloma."

The RAG response captures the key therapeutic insight that this combination approach could "overcome resistance mechanisms involving MCL-1," which aligns closely with the ground truth explanation that the strategy "could potentially overcome intrinsic resistance mechanisms in solid tumors that are less dependent on BCL-2 but still rely on MCL-1 for survival." This mechanistic understanding is crucial for clinical translation, as it identifies the specific molecular rationale for why the combination might succeed where single-agent BCL-2 inhibition fails.

In contrast, the standard GPT-4o response, while comprehensive and well-structured, provides a generic framework that could apply to virtually any combination therapy discussion. The response covers broad categories such as "mechanistic synergy," "clinical efficacy," and "resistance mechanisms" but fails to reference the specific NOXA-mediated pathway that makes this particular combination therapeutically promising. Critical omissions include the ISR activation, ATF3/ATF4 transcriptional regulation, and the specific role of MCL-1 inhibition in overcoming resistance.

The standard response does acknowledge that "solid tumors might not be as dependent on BCL-2 as hematologic malignancies" and mentions reliance on "other anti-apoptotic

Domain-specific question:
What are the implications of the findings regarding the combinatorial use of ABT-199 and proteasome inhibitors for treating solid tumors compared to hematologic malignancies?

AgentD (w/ RAG):

The findings indicate that combining ABT-199 (venetoclax) with proteasome inhibitors (PIs) could significantly impact the treatment of both solid tumors and hematologic malignancies. In solid tumors, such as soft tissue sarcoma, this combination synergistically induces apoptosis by activating the pro-apoptotic protein NOXA, which inhibits the anti-apoptotic protein MCL-1, enhancing ABT-199's efficacy. This dual action—direct BCL-2 inhibition and indirect MCL-1 inhibition—suggests effectiveness in solid tumors overexpressing these proteins. In hematologic malignancies, where ABT-199 is already effective, particularly in chronic lymphocytic leukemia and multiple myeloma, combining it with PIs could improve outcomes by overcoming resistance mechanisms involving MCL-1. These findings support further clinical evaluation of this combination in various cancers, potentially expanding therapeutic options for both solid and hematologic malignancies.

Standard GPT4o:

The findings on the combinatorial use of ABT-199 (venetoclax) and proteasome inhibitors have several implications for the treatment of solid tumors compared to hematologic malignancies. Here's a breakdown:

1. Mechanistic Synergy:
Hematologic Malignancies: ABT-199, a BCL-2 inhibitor, is particularly effective because these cancers often rely heavily on the BCL-2 pathway for survival. When combined with proteasome inhibitors, which block protein degradation, the apoptotic pathways are further enhanced, leading to increased cancer cell death.
Solid Tumors: These cancers might not be as dependent on BCL-2 as hematologic malignancies. However, the combination could still disrupt protein homeostasis and induce apoptosis by targeting multiple pathways.

2. Clinical Efficacy:
Hematologic Malignancies: Clinical trials have shown promising results due to the high BCL-2 expression. The combinatorial approach can enhance responses in resistant cases.
Solid Tumors: The efficacy might be more variable due to the diverse nature of these tumors and their reliance on other anti-apoptotic proteins like MCL-1 or BCL-XL.
Resistance Mechanisms:

Hematologic Malignancies: Resistance can develop through mutations or upregulation of alternative survival pathways.
Solid Tumors: The diverse genetic landscape means multiple resistance mechanisms could limit effectiveness.

3. Toxicity and Side Effects:
Combining ABT-199 with proteasome inhibitors can potentially increase toxicity, necessitating careful dose optimization and monitoring in both cancer types.

4. Biomarker Development:
Identifying biomarkers to predict response can help tailor the combination therapy more effectively, particularly in solid tumors where response rates might be heterogeneous.

5. Future Research Directions:
Continued research is needed to understand the specific tumor types and settings where this combination is most beneficial, and to develop strategies to overcome resistance in solid tumors.
Overall, while the combination has shown efficacy in hematologic cancers, its application in solid tumors requires a nuanced understanding of tumor biology and careful clinical evaluation.

Ground Truth (Human-Annotated):
Solid tumors have shown to be less dependent on BCL-2 as compared to leukemias. This might explain the limited efficacy of BCL-2 inhibitors such as ABT-199 in most solid tumors. While solid tumors are generally less BCL-2 dependent than hematological malignancies, the combination of ABT-199 and proteasome inhibition may overcome this limitation by enhancing NOXA expression and blocking its degradation, thereby targeting both BCL-2 and MCL-1. Our results indicate that the combination of ABT-199 and proteasome inhibition could also be active in various solid malignancies by simultaneously inhibiting BCL-2 and MCL-1. The presented mechanism suggests that dual targeting of BCL-2 and MCL-1 could represent a therapeutic strategy not only for hematological malignancies but also for solid tumors.

Figure S1: AgentD response demonstrating domain-specific question answering capabilities. The question extrapolates beyond the direct scope of the source article.

proteins like MCL-1 or BCL-XL," but it lacks the mechanistic foundation to explain how the combination specifically addresses these dependencies. This represents a missed opportunity to provide actionable clinical insights based on the underlying biology.

Furthermore, while the standard response discusses general considerations such as toxicity management and biomarker development, it does so without the mechanistic context that would guide these clinical decisions. The RAG response, by contrast, grounds its recommendations in the specific findings about NOXA upregulation and MCL-1 inhibition, providing a more targeted foundation for clinical development.

This evaluation demonstrates that even for questions that extend beyond the immediate experimental scope of the reference literature, RAG-augmented responses maintain superior clinical relevance by preserving the mechanistic foundation that underlies therapeutic potential. The ability to connect specific molecular mechanisms to broader therapeutic applications represents a critical advantage for biomedical question answering systems, particularly in translational research contexts where mechanistic understanding directly informs clinical strategy.

To further test the system's generalizability, we evaluated AgentD on a study describing resistance pathways to the EGFR inhibitor osimertinib in non-small-cell lung cancer (NSCLC).[S2] Here, we designed a three-part question sequence to assess whether the model could (i) enumerate all known resistance mechanisms, (ii) distinguish EGFR-dependent mechanisms (e.g., tertiary mutations and amplification events), and (iii) explain EGFR-independent mechanisms involving bypass signaling and lineage transformation. In this evaluation, the RAG-augmented model retrieved the appropriate literature and produced coherent, hierarchically structured responses, correctly classifying each mechanistic category and describing the molecular basis of resistance in Figure S2, S3. By contrast, GPT-4o generated lengthy but undifferentiated lists of mechanisms without distinguishing between dependent and independent processes, resulting in lower factual clarity. These findings indicate that retrieval grounding improves conceptual organization and mechanistic specificity,

particularly in cases where questions require multi-step reasoning rather than factual recall.

Domain-specific question:
List the possible mechanisms by which non-small-cell lung cancers develop resistance to the EGFR inhibitor Osimertinib.

AgentD (w/ RAG):
Resistance to osimertinib in non-small-cell lung cancer (NSCLC) can occur through various mechanisms, categorized into EGFR-dependent and EGFR-independent mechanisms. EGFR-dependent mechanisms include tertiary EGFR mutations like C797S, EGFR amplifications, and loss of the T790M mutation. EGFR-independent mechanisms involve MET and HER2 amplifications, activation of RAS-MAPK and PI3K pathways, histological transformations to small-cell lung cancer or squamous cell carcinoma, epithelial-to-mesenchymal transition (EMT), and novel gene fusions or mutations in genes like BRAF, ALK, and RET. These mechanisms highlight the complexity and heterogeneity of resistance to osimertinib.

Standard GPT4o:
Non-small-cell lung cancer (NSCLC) can develop resistance to the epidermal growth factor receptor (EGFR) inhibitor osimertinib through several mechanisms. Some of the well-documented mechanisms include:
Secondary EGFR Mutations: The development of additional mutations in the EGFR gene, such as the C797S mutation, can prevent osimertinib from binding effectively to the EGFR protein.
MET Amplification: This involves the amplification of the MET gene, which can activate alternative signaling pathways, bypassing the inhibited EGFR pathway and sustaining tumor growth.
HER2 Amplification: Similar to MET, HER2 (also known as ERBB2) amplification can activate downstream signaling pathways and contribute to resistance.
Histological Transformation: In some cases, the tumor may undergo histological transformation, such as shifting from adenocarcinoma to small cell lung cancer (SCLC) phenotype, which is less responsive to osimertinib.
KRAS Mutations: The emergence of KRAS mutations can activate the RAS/RAF/MEK/ERK signaling pathway, promoting cancer cell survival and proliferation.
PIK3CA Mutations: Mutations in the PIK3CA gene can lead to activation of the PI3K/AKT signaling pathway, allowing cancer cells to bypass the inhibited EGFR signal.
Activation of Alternative Pathways: Other pathways, such as AXL activation, can also be involved in resistance, enabling cancer cells to continue proliferating despite EGFR inhibition.
EGFR-independent Mechanisms: Some tumors develop resistance through mechanisms that are independent of EGFR alterations, which might involve changes in the tumor microenvironment or other cell survival signaling pathways.
Understanding these mechanisms is crucial for the development of next-line therapies and combination strategies to overcome or delay resistance to osimertinib in NSCLC patients.

Ground Truth (Human-Annotated):
Resistance to osimertinib in EGFR-mutated non-small-cell lung cancer arises through a range of molecular alterations that can be broadly classified as EGFR-dependent or EGFR-independent mechanisms.
EGFR-dependent mechanisms include the emergence of tertiary EGFR mutations, most commonly C797S, L792H/F, G796R/S/D, L718Q, G719A, G724S, and exon 20 insertions, which interfere with covalent binding or alter the drug-binding pocket, as well as EGFR gene amplification or increased EGF expression that restore downstream signalling.
EGFR-independent mechanisms involve activation of bypass and downstream pathways such as MET amplification, HER2 amplification, RAS–MAPK activation, PI3K pathway activation, cell-cycle gene alterations, and oncogenic fusions (e.g., RET, NTRK, BRAF).
Additional processes include histologic transformation (e.g., small-cell conversion), epithelial-to-mesenchymal transition (EMT), and activation of alternative kinases such as AXL, FAK, or FGFR1.

Figure S2: AgentD response demonstrating retrieval-grounded reasoning for a mechanistic question on EGFR inhibitor resistance. The RAG-based model retrieves relevant literature and organizes the identified mechanisms into EGFR-dependent and EGFR-independent categories, maintaining a coherent hierarchical structure compared to the unorganized GPT-4o response.

To complement these qualitative comparisons, we quantitatively assessed response alignment using RoBERTa[S3]-based BERTScore. As summarized in Table S1, AgentD with RAG achieved consistently higher precision, recall, and F1-scores than the non-RAG baseline, closely mirroring the performance trend observed with SciBERT in the main text. These

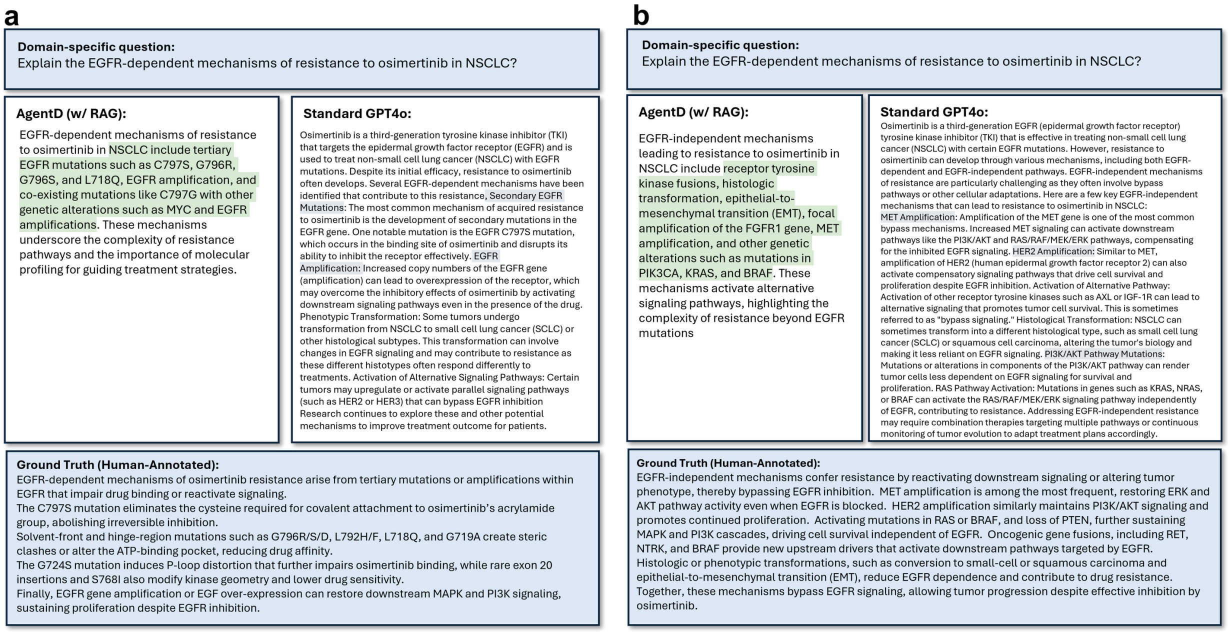

Figure S3: AgentD responses to detailed follow-up questions on EGFR-dependent (left) and EGFR-independent (right) resistance mechanisms. The RAG-augmented model preserves mechanistic coherence and factual separation between categories, whereas GPT-4o produces overlapping, less structured explanations.

results confirm that the observed improvements in factual grounding and contextual alignment are robust to the choice of embedding encoder and not dependent on model-specific representations.

Table S1: Comparison of BERTScores between AgentD (with RAG) and vanilla GPT-4o (without RAG) using RoBERTa embeddings. Values are averaged across six question–answer pairs and complement the SciBERT-based results in Table 2.

Method	Precision	Recall	F1-score
AgentD (GPT-4o, w/ RAG)	0.892 ± 0.015	0.876 ± 0.030	0.884 ± 0.018
Vanilla GPT-4o (w/o RAG)	0.847 ± 0.009	0.865 ± 0.017	0.872 ± 0.040
AgentD (DeepSeek-Chat, w/ RAG)	0.872 ± 0.033	0.887 ± 0.019	0.879 ± 0.022
Vanilla DeepSeek-Chat (w/o RAG)	0.838 ± 0.014	0.874 ± 0.040	0.847 ± 0.011

In addition, we implemented control over the selection of target papers by applying filtering criteria based on citation ranking and publication year to retrieve more relevant and reliable sources for the RAG approach. We then assessed the impact of these retrieval strategies (citation-based and publication-year-based) on question–answering performance

by comparing RAG-augmented AgentD with a vanilla GPT-4o baseline. Citation-ranked retrieval was implemented by sorting Semantic Scholar search results in descending order of citation count through the API's built-in ranking parameter. This evaluation was conducted using three BCL-2–specific mechanistic question–answer pairs corresponding to the queries analyzed in Figure 3a,b and Supplementary Figure S2, allowing us to isolate retrieval effects without introducing additional biological variability. Performance was quantified using SciBERT-based BERTScore, with the results summarized in Table S2.

Table S2: Effect of retrieval filtering strategies on RAG-based question answering performance using SciBERT embeddings. Results are evaluated on three BCL-2–specific mechanistic question–answer pairs.

**Method**	**Precision**	**Recall**	**F1-score**
Vanilla GPT-4o	0.617 ± 0.018	0.695 ± 0.019	0.653 ± 0.012
AgentD (GPT-4o, w/ RAG; minimal)	0.700 ± 0.041	0.729 ± 0.038	0.714 ± 0.039
AgentD (GPT-4o, w/ RAG; citation-ranked)	0.640 ± 0.007	0.648 ± 0.022	0.644 ± 0.013
AgentD (GPT-4o, w/ RAG; year-filtered, $\geq$2022)	0.690 ± 0.035	0.711 ± 0.042	0.700 ± 0.038

Across all configurations, RAG augmentation consistently outperformed vanilla GPT-4o, demonstrating improved grounding and contextual alignment with the reference literature. Year-filtered retrieval (publication year $\geq$ 2022) yielded comparable performance, suggesting that recency constraints do not substantially degrade answer quality for well-defined mechanistic queries. In contrast, citation-ranked retrieval resulted in lower precision and F1-score, reflecting limited coverage for narrowly scoped mechanistic questions. Highly cited articles do not always contain the specific pathway-level details required for fine-grained reasoning. In particular, for the query in Figure 3b, the citation-ranked configuration retrieved highly cited studies that lacked the necessary mechanistic information, causing the agent to explicitly indicate insufficient evidence rather than generate an unsupported response. Overall, these results demonstrate that RAG-based grounding improves factual alignment relative to vanilla GPT-4o, while overly restrictive retrieval filters can reduce performance for pathway-specific mechanistic reasoning.

S2 Failure Cases with Claude-3.7-Sonnet

In the system prompt, the model is instructed to return the original SMILES after three failed attempts, yet Claude-3.7-Sonnet often becomes stuck in an apparent infinite loop (Figure S4).

```
....
Thought: I need to analyze the properties of the given molecule to identify its weaknesses
as a drug candidate targeting BCL-2. Then I'll propose a modification to address the most
critical weakness.

First, let me understand the original SMILES:
Cc1c(CCC(C)C)c(=O)oc2cc(OS(=O)(=O)c3ccc(Cl)cc3)ccc12

Looking at the properties, I notice several potential issues:
1. Poor absorption: Caco-2 (logPaap) is -5.27, which is very low
2. Blood-Brain Barrier penetration is -2.14, which might be undesirable for a non-CNS target
3. Multiple CYP inhibitions (1A2, 2C19, 2C9) and substrate status (2C9, 3A4) - potential for drug interactions
4. Several toxicity concerns: Toxic to bees, crustaceans, eye irritation, liver injury II, micronucleos
5. High lipophilicity: Log(P) is 5.47, which is above the ideal range for oral drugs (typically 2-5)

The most critical weakness appears to be the high lipophilicity (Log(P) = 5.47), which
likely contributes to the toxicity issues and may affect bioavailability. For BCL-2 targeting,
we don't need BBB penetration, so I'll focus on reducing lipophilicity.

I'll modify the structure by replacing the lipophilic chlorine atom with a more polar group,
such as a hydroxyl group, and possibly modify the alkyl chain to reduce lipophilicity.

Action: check_smiles_validity
Action Input: Cc1c(CCC(C)C)c(=O)oc2cc(OS(=O)(=O)c3ccc(O)cc3)ccc12

Observation: Valid SMILES

Thought: I need to analyze the properties of the given molecule to identify its weaknesses
as a drug candidate targeting BCL-2. Then I'll propose a modification to address the most
critical weakness.

Looking at the properties, I notice several potential issues:
1. Poor absorption: Caco-2 (logPaap) is -5.27, which is very low
2. Multiple CYP inhibitions (1A2, 2C19, 2C9) and substrate status (2C9, 3A4)
3. Several toxicity concerns: Toxic to bees, crustaceans, eye irritation, liver injury II, micronucleos
4. High lipophilicity: Log(P) is 5.47, which is above the ideal range for oral drugs

The most critical weakness appears to be the high lipophilicity (Log(P) = 5.47). I'll replace
the chlorine atom with a hydroxyl group.

Action: check_smiles_validity
Action Input: Cc1c(CCC(C)C)c(=O)oc2cc(OS(=O)(=O)c3ccc(O)cc3)ccc12

Observation: Valid SMILES
....
```

Figure S4: Failure cases in Claude-3.7-Sonnet refinement showing repeated iteration loops.

S3 ADMET Properties

Table S3: Complete list of ADMET properties predicted for all compounds in this study.

Category	Property	Description
**Absorption**	Caco-2 Permeability (logPaap)	Permeability across Caco-2 cell monolayers
	Human Intestinal Absorption	Fraction absorbed in human intestine
	Human Oral Bioavailability (20%)	Probability of achieving >20% oral bioavailability
	Human Oral Bioavailability (50%)	Probability of achieving >50% oral bioavailability
	MDCK Permeability	Madin-Darby Canine Kidney cell permeability
	P-Glycoprotein Inhibitor	Inhibition of P-glycoprotein efflux pump
	P-Glycoprotein Substrate	Substrate of P-glycoprotein efflux pump
	Skin Permeability	Dermal absorption coefficient
**Distribution**	Blood-Brain Barrier Penetration	CNS penetration capability
	Blood-Brain Barrier (log BB)	Blood-brain barrier partition coefficient
	Fraction Unbound (Human)	Unbound fraction in human plasma

Continued on next page

Table S3 – *Continued from previous page*

Category	Property	Description
	Plasma Protein Binding	Extent of protein binding in plasma
	Volume of Distribution	Steady-state volume of distribution
**Metabolism**	BCRP Substrate	Breast Cancer Resistance Protein substrate
	CYP1A2 Inhibitor	Cytochrome P450 1A2 inhibition
	CYP1A2 Substrate	Cytochrome P450 1A2 substrate
	CYP2C19 Inhibitor	Cytochrome P450 2C19 inhibition
	CYP2C19 Substrate	Cytochrome P450 2C19 substrate
	CYP2C9 Inhibitor	Cytochrome P450 2C9 inhibition
	CYP2C9 Substrate	Cytochrome P450 2C9 substrate
	CYP2D6 Inhibitor	Cytochrome P450 2D6 inhibition
	CYP2D6 Substrate	Cytochrome P450 2D6 substrate
	CYP3A4 Inhibitor	Cytochrome P450 3A4 inhibition
	CYP3A4 Substrate	Cytochrome P450 3A4 substrate
	OATP1B1 Substrate	Organic Anion Transporting Polypeptide 1B1
	OATP1B3 Substrate	Organic Anion Transporting Polypeptide 1B3
**Excretion**	Clearance	Total body clearance
	Half-Life	Elimination half-life
	OCT2 Substrate	Organic Cation Transporter 2 substrate
**Toxicity**	AMES Mutagenicity	Bacterial mutagenicity test

Continued on next page

Table S3 – *Continued from previous page*

Category	Property	Description
	Avian Toxicity	Acute toxicity to birds
	Bee Toxicity	Acute toxicity to honeybees
	Bioconcentration Factor	Bioaccumulation potential
	Biodegradation	Environmental biodegradability
	Carcinogenicity	Carcinogenic potential
	Crustacean Toxicity	Acute toxicity to crustaceans
	Daphnia Toxicity	Acute toxicity to *Daphnia magna*
	Eye Corrosion	Severe eye damage potential
	Eye Irritation	Eye irritation potential
	Fathead Minnow Toxicity	Acute toxicity to *Pimephales promelas*
	Hepatotoxicity (DILI)	Drug-induced liver injury
	Hepatotoxicity (Alternative)	Alternative liver injury prediction
	Maximum Tolerated Dose	Highest non-toxic dose
	Micronucleus Test	Chromosomal damage potential
	Nuclear Receptor AhR	Aryl hydrocarbon receptor activation
	Nuclear Receptor AR	Androgen receptor binding
	Nuclear Receptor AR-LBD	Androgen receptor ligand binding domain
	Nuclear Receptor Aromatase	Aromatase enzyme inhibition
	Nuclear Receptor ER	Estrogen receptor binding

Continued on next page

Table S3 – *Continued from previous page*

Category	Property	Description
	Nuclear Receptor ER-LBD	Estrogen receptor ligand binding domain
	Nuclear Receptor GR	Glucocorticoid receptor binding
	Nuclear Receptor PPAR-γ	Peroxisome proliferator-activated receptor γ
	Nuclear Receptor TR	Thyroid receptor binding
	Rat Acute Toxicity	Acute oral toxicity in rats
	Rat Chronic Toxicity	Chronic oral toxicity in rats
	Respiratory Toxicity	Respiratory system toxicity
	Skin Sensitization	Dermal sensitization potential
	Stress Response ARE	Antioxidant response element activation
	Stress Response ATAD5	ATAD5 genotoxicity pathway
	Stress Response HSE	Heat shock response element
	Stress Response MMP	Mitochondrial membrane potential
	Stress Response p53	p53 tumor suppressor pathway
	Tetrahymena pyriformis	Acute toxicity to *T. pyriformis*
	hERG Inhibition	Human ether-à-go-go-related gene K+ channel
**General**	Boiling Point	Boiling point temperature
	Hydration Free Energy	Free energy of hydration
	log D (pH 7.4)	Distribution coefficient at physiological pH

Continued on next page

Table S3 – *Continued from previous page*

Category	Property	Description
	log P	Octanol-water partition coefficient
	log S	Aqueous solubility
	log Vapor Pressure	Vapor pressure
	Melting Point	Melting point temperature
	pKa (Acidic)	Acid dissociation constant
	pKa (Basic)	Base dissociation constant
	pKd (Acidic)	Alternative acid dissociation constant

S4 Identified Unfavorable Drug Properties

The molecular optimization process focuses on identifying unfavorable properties and refining SMILES to improve their ADMET profiles. Table S4 summarizes the distribution of properties flagged by AgentD across optimization rounds, grouped by ADMET categories.

An observed limitation is the agent's frequent selection of $\log P_{\mathrm{app}}$ as a risk factor—despite it being just one among 75 possible properties (67 ADMET-related, 7 general physicochemical, and 1 binding affinity). This suggests a selection bias, potentially due to the ordering of properties in the input dictionary. Since $\log P_{\mathrm{app}}$ appears near the top of the dictionary entry, the agent may disproportionately attend to it during its reasoning process. Future iterations can address this by randomizing property order or introducing attention calibration techniques.

Table S4: Distribution of weakness properties after 1st and 2nd round of SMILES optimization. The weakness property represents the most critical ADMET deficiency targeted for improvement.

**ADMET Category**	**Specific Property**	**1st Round (%)**	**2nd Round (%)**
Absorption	Caco-2 (logPaap)	59.6	69.5
Toxicity	AMES Mutagenesis	14.1	7.4
	Liver Injury I (DILI)	5.1	4.2
	hERG Blockers	4.0	2.1
	Carcinogenesis	3.0	1.1
General Properties	logP	5.1	3.2
	logS	1.0	–
Other	Absorption/Human Oral Bioavailability (20% and 50%), Invalid SMILES, and other general entries.	6.0	8.4
**Total Entries**		**99**	**95**

S5 JAK-2 for Myelofibrosis

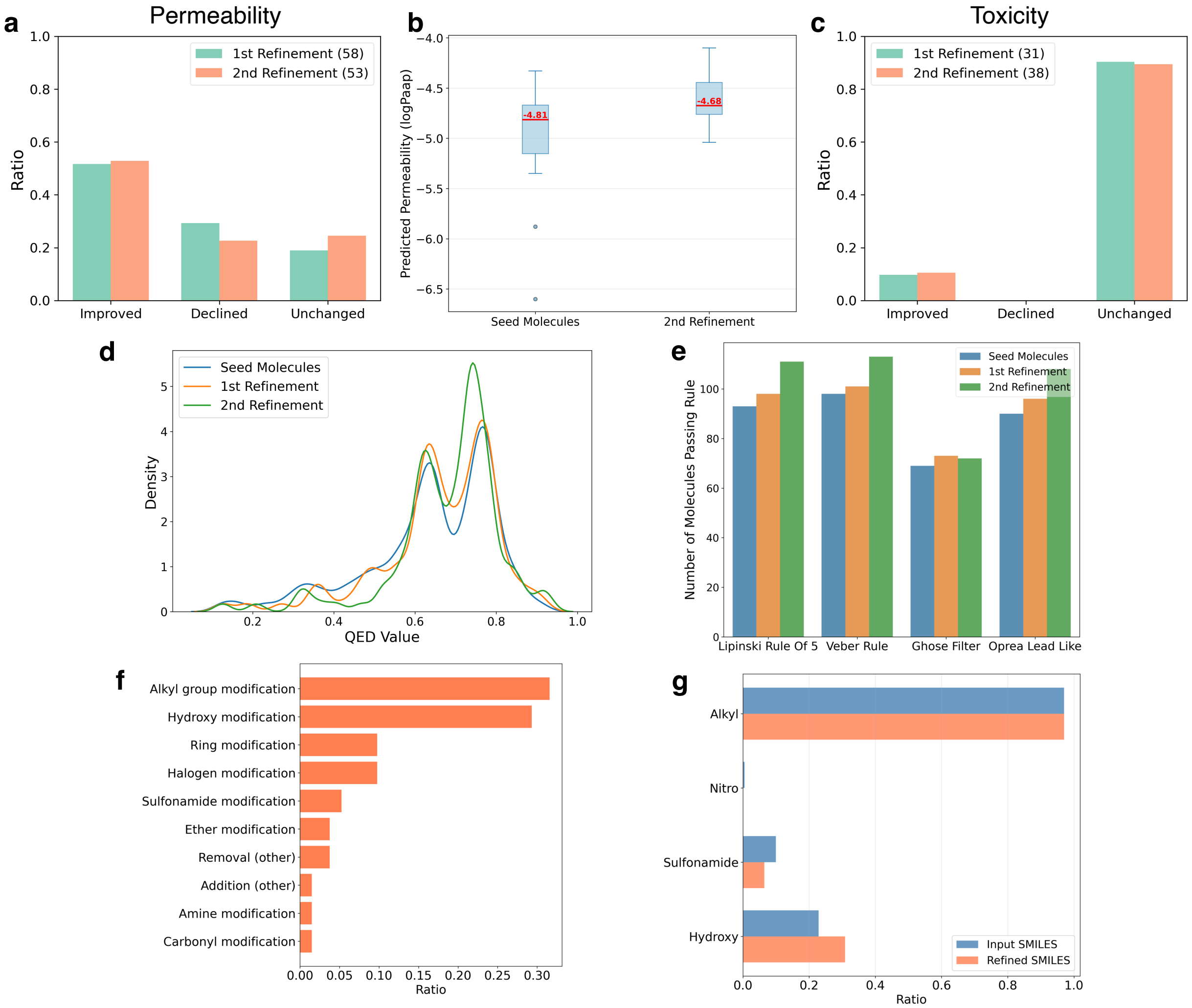

Figure S5: Property-aware molecular refinement results for JAK2. **a** Proportion of molecules with improved, declined, or unchanged permeability (logP_{app}). **b** Boxplot of logP_{app} for entries showing improvement. **c** Molecular refinement patterns for molecules with improved permeability. **d** QED distribution shifts across iterations. **e** Counts of molecules satisfying empirical drug-likeness rules. **f** Fraction of molecules modified for permeability. **g** Functional-group frequencies in input and refined molecules across iterations.

The drug-likeness of the molecule pool increases over refinement iterations (Figure S5a–c), following the same general trends observed in the primary BCL-2 case study (Figure 6), including increases in predicted permeability and toxicity. However, the extent of improvement is more modest for JAK-2. The mean QED increases only from 0.64 to 0.68, and the

proportion of molecules with QED > 0.6 increases from 71.0% to 84.4% (Figure S5d). In contrast, for BCL-2 the mean QED rose from 0.47 to 0.57, and the fraction above QED 0.6 nearly doubled (23.0% $\rightarrow$ 45.7%). The rule-based evaluations also show a distinct pattern. Unlike the BCL-2 case, JAK-2 exhibits only marginal improvement under the Ghose filter, while Lipinski's Rule of Five and the Veber filter show more substantial gains (Figure S5e). These differences highlight molecule-dependent refinement behavior and the intrinsic complexity of SMILES-based molecular optimization, where multiple properties interact and trade off in non-linear ways.

S6 Thrombin for Thrombosis

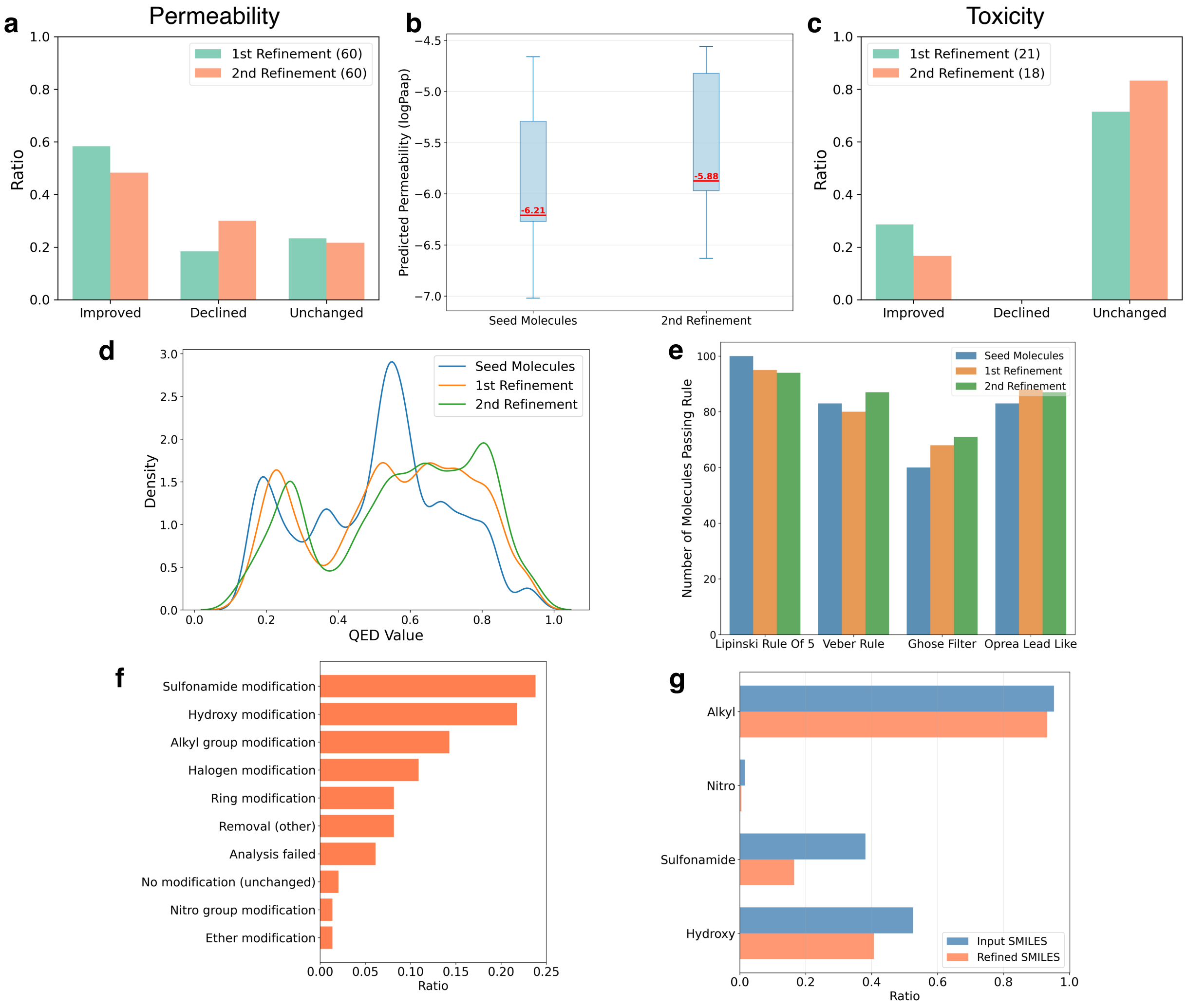

Figure S6: Property-aware molecular refinement results for Thrombin. **a** Proportion of molecules with improved, declined, or unchanged permeability ($\log P_{app}$). **b** Boxplot of $\log P_{app}$ for entries showing improvement. **c** Molecular refinement patterns for molecules with improved permeability. **d** QED distribution shifts across iterations. **e** Counts of molecules satisfying empirical drug-likeness rules. **f** Fraction of molecules modified for permeability. **g** Functional-group frequencies in input and refined molecules across iterations.

The Thrombin case also demonstrates clear improvements in drug-likeness following iterative refinement, exhibiting a trend more similar to the primary BCL-2 case study. The mean QED increases from 0.51 in the seed pool to 0.57 after the second refinement. Likewise, the proportion of molecules with QED $>$ 0.6 rises substantially from 30.0% to 51.1%,

indicating that the refinement process effectively shifts the pool toward more drug-like chemical space.The rule-based evaluation patterns further reinforce this behavior. Similar to the BCL-2 results, the Thrombin set shows notable improvements under the Ghose filter, reflecting enhanced balance in physicochemical properties such as molecular weight and logP. In contrast, Lipinski's Rule of Five metrics exhibit slight deterioration, suggesting mild increases in features such as molecular size or hydrogen-bond counts that are penalized in this rule set.

S7 Empirical Filters

The listed properties were calculated using RDKit with molecular SMILES as input. Note that these rules serve as illustrative examples and are not the definitive criteria for selecting candidates for 3D structure generation.

In addition to the four rules described in the main manuscript, we also include the Rule of Three in Table S5. The Rule of Three was introduced as a guideline for fragment-based drug discovery (FBDD) to identify suitable fragments for screening libraries.[S4] Unlike drug-likeness rules such as Lipinski's Rule of Five or Veber's rule, or lead-likeness filters such as Oprea's, the Rule of Three is tailored for much smaller chemical fragments that serve as starting points in fragment-based approaches. In contrast to drug-likeness and lead-likeness rules, which focus on compounds closer to optimized drugs or leads, the Rule of Three prioritizes low molecular complexity and high chemical tractability, ensuring fragments can be readily optimized in subsequent stages of drug development.

Table S5: Summary of rule-based empirical filters and their physicochemical criteria.

Rule	Criterion	Threshold / Range	Pharmacological Rationale
Lipinski's Rule of Five[S5,S6]	Molecular Weight (MW)	$\leq$ 500 Da	High molecular weight is associated with poor intestinal absorption due to size-related transport limitations.
	logP	≤ 5	High lipophilicity (logP $\leq$ 5) correlates with poor aqueous solubility and passive permeability.
	H-bond Donors (HBD)	≤ 5	Excessive H-bond donors increase polarity, reducing membrane permeability.
	H-bond Acceptors (HBA)	≤ 10	Too many acceptors increase polarity, hindering passive diffusion.
	Rotatable Bonds (RB)	$\leq$ 10 rotatable bonds	High flexibility and polar surface area reduce the likelihood of oral activity.
Veber Rule[S6]	TPSA	$\leq$ 140 Å^2	High polar surface area decreases oral bioavailability by reducing passive diffusion.
	Rotatable Bonds (RB)	≤ 10	Excess flexibility increases entropy, reducing oral bioavailability and metabolic stability.
Ghose Filter[S7]	Molecular Weight (MW)	160–480 Da	Balances molecular size for optimal binding and permeability.
	logP	-0.4 to 5.6	Ensures moderate lipophilicity for both solubility and membrane crossing.
	Molar Refractivity (MR)	40–130	Captures molecular volume and polarizability, influencing receptor binding.
	Atom Count	20–70	Reflects a size range favorable for drug-likeness and synthetic accessibility.
Rule of Three (Ro3)[S8]	Molecular Weight (MW)	$<$ 300 Da	Smaller fragments are preferred in fragment-based drug discovery for lead optimization.
	logP	≤ 3	Low lipophilicity promotes solubility in fragment-like compounds.
	H-bond Donors (HBD)	≤ 3	Reduces polarity, supporting fragment permeability and binding.
Oprea Lead-like Filter[S9]	Molecular Weight (MW)	200–450 Da	Ideal size range for optimization into drug-like compounds.
	logP	-1 to 4.5	Moderate hydrophobicity for balanced solubility and permeability.
	Rotatable Bonds (RB)	≤ 8	Lower conformational entropy improves binding efficiency.
	Aromatic Rings	≤ 4	Limits excessive aromaticity, which can affect solubility and toxicity.
	H-bond Donors (HBD)	≤ 5	Controls molecular polarity and improves membrane permeability.
	H-bond Acceptors (HBA)	≤ 8	Keeps polarity within bounds for favorable pharmacokinetics.

S8 Structure Generation Examples

Using the illustrative criteria described in the main manuscript, namely (i) satisfying Oprea's lead-likeness filter, (ii) passing at least two of the three drug-likeness rules (Lipinski's Rule of Five, Veber's rule, and Ghose's filter), (iii) achieving a QED score above 0.55, and (iv) having a predicted pK_d value greater than 6.0, three molecules remained after filtering from a total of 289 molecules generated through SMILES generation and property aware refinement. Among these, the molecule O=C(NS(=O)(=O)c1ccc(N2CCNCC2)c(C)c1)c1ccc(N2CCOCC2)c(Cl)c1 was selected as an example for three dimensional structure generation.

- **O=C(NS(=O)(=O)c1ccc(N2CCNCC2)c(O)c1)c1ccc(N2CCOCC2)c(Cl)c1**
  - Source: Mol2Mol
  - Lipinski: True, Veber: True, Ghose: False, Oprea: True
  - QED: 0.589, Affinity (pK_d): 6.194
- **O=C(NS(=O)(=O)c1ccc(N2CCNCC2)c(C)c1)c1ccc(N2CCOCC2)c(Cl)c1**
  - Refinement
  - Lipinski: True, Veber: True, Ghose: True, Oprea: True
  - QED: 0.679, Affinity (pK_d): 6.182
- **O=C(NS(=O)(=O)c1ccc(N2CCOCC2=O)c(Cl)c1)c1ccc(N2CCOCC2)c(Cl)c1**
  - Source: Refinement
  - Lipinski: True, Veber: True, Ghose: False, Oprea: True
  - QED: 0.653, Affinity (pK_d): 6.077

Because the molecules shown above are very similar, all sharing the common starting SMILES fragment 'O=C(NS(=O)(=O)c1ccc(N2CC', we included additional examples for visualization. For the molecules presented in Figure S7, we applied stricter rule-based filters

and slightly more lenient pK_d thresholds than those used in the main manuscript. Specifically, molecules were selected if they passed more than four rule-based filters, had a QED score above 0.6, and a predicted pK_d greater than 5.5. From the set of molecules that underwent two rounds of refinement, three examples were randomly chosen for illustration. Again, this does not aim for practical application, but only to select a few examples for visualization.

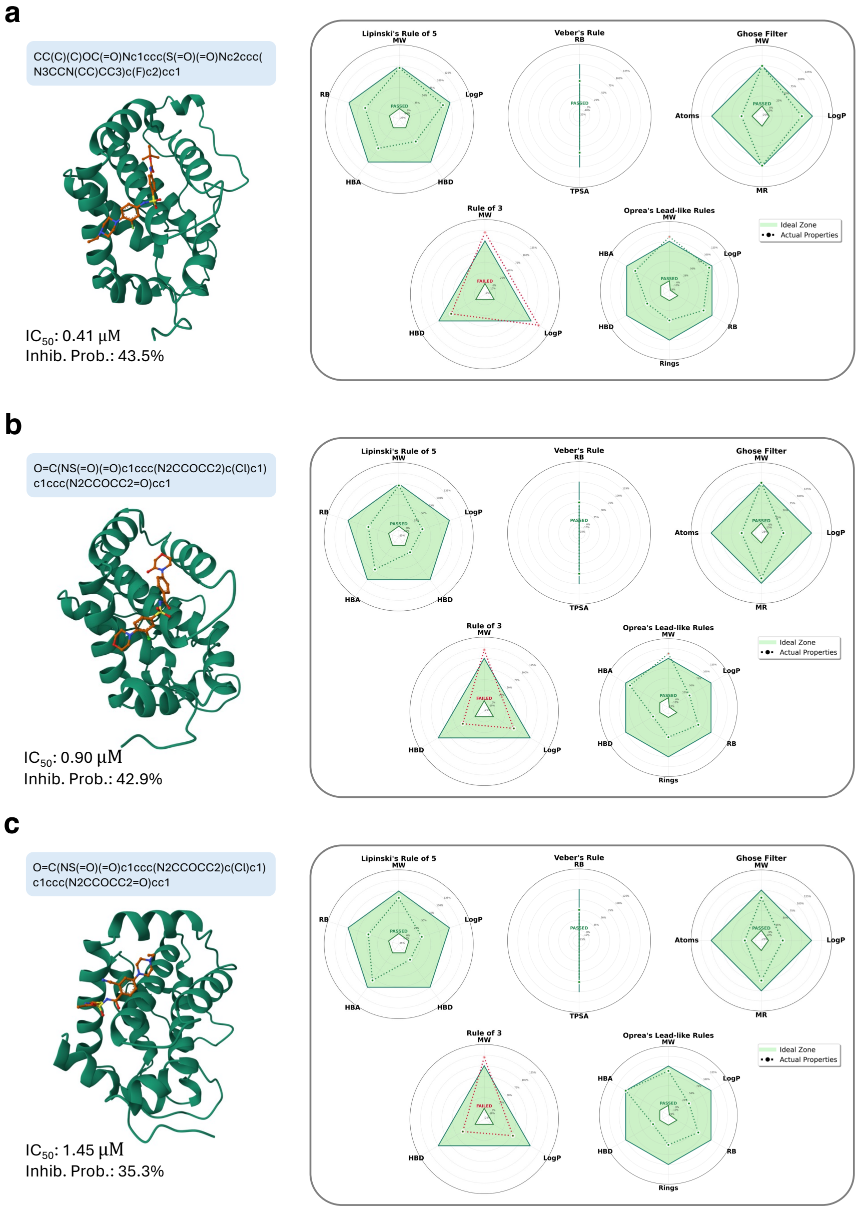

Figure S7: Additional examples of protein–ligand complex generation and evaluation.

S9 LLM-based Molecule Optimization Approaches

Evaluating AgentD against existing LLM-driven agentic systems presents inherent challenges, largely because these systems differ substantially in architecture, design objectives, and evaluation methodologies. While several agents operate in molecular design or autonomous scientific reasoning, their workflows are generally not aligned with the end-to-end optimization paradigm that AgentD implements. As a result, establishing a standardized, quantitative benchmark across systems is often infeasible or structurally invalid. Accordingly, we restrict quantitative comparisons to frameworks that share comparable optimization objectives and evaluation semantics with AgentD.

Most existing agents fall into one of two categories: (i) single-step property computation tools or (ii) generalist orchestrators that coordinate tool calls without performing internal chemical reasoning. For instance, CACTUS functions solely as a descriptor calculator, providing molecular properties such as molecular weight or LogP without any generative or refinement capabilities. DrugPilot, while more sophisticated, primarily acts as a dialogue-based orchestrator that forwards optimization tasks to a single external, domain-specific model; its performance is evaluated almost exclusively on tool-calling reliability rather than molecular design quality. SciToolAgent offers broad scientific coverage through knowledge-graph-based tool chaining, but it is intended as a general-purpose scientific assistant rather than a specialized pipeline for drug discovery.

Among existing systems, dZiner offers the closest conceptual point of comparison because it also employs an iterative molecular refinement loop. However, the underlying design objectives differ markedly. dZiner focuses on optimizing binding affinity, using Quantitative Estimate of Drug-likeness (QED) and synthetic accessibility (SA) only as auxiliary metrics. AgentD executes self-diagnostic molecular refinement, integrating structural validity checks.

We demonstrated the differing objectives of dZiner and AgentD using ten representative molecules, including those shown in Figure 5 of the main manuscript. As illustrated in Figure S8, dZiner does not improve QED scores; instead, its full-iteration outputs tend

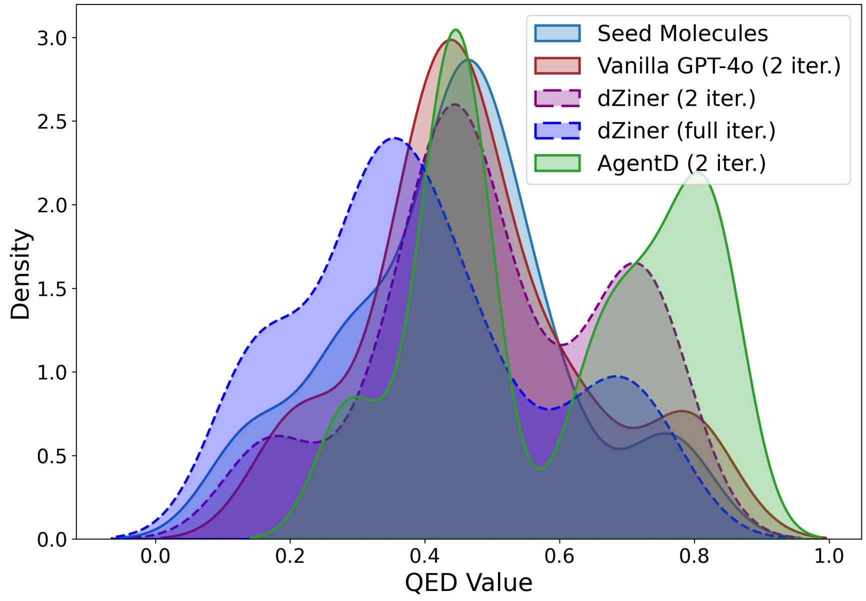

Figure S8: Comparison of QED distributions for ten representative molecules across four design strategies: the original seed molecules, vanilla GPT-4o after two refinement iterations, dZiner after two iterations, and AgentD (GPT-4o) using its two-iteration workflow. For consistency, only the first two iterations of dZiner and vanilla GPT-4o are included to match the two-iteration refinement cycle used by AgentD.

to shift toward lower QED relative to the initial seed molecules and their second-iteration intermediates. This outcome is expected, given that dZiner optimizes strictly for binding affinity. The divergence between dZiner and AgentD highlights a fundamental difference in design philosophy.

Table S6: Comparison of cost, token usage, and API requests per sample for different models.

**Model**	**Avg. Cost per Sample ($)**	**Avg. Token Usage per Sample**	**Avg. API Requests per Sample**
Vanilla GPT-4o (2 iter.)	$0.003	318.7	41.7
AgentD (w/ GPT-4o; 2 iter.)	$0.022	9087.4	6.6
dZiner (w/ GPT-4o; full iter.)	$0.533	193875.7	1

Beyond objective differences, the systems exhibit marked contrasts in computational cost. Table S6 summarizes the average token consumption, monetary cost, and number of API requests per sample for Vanilla GPT-4o (two-step direct prompting), AgentD using GPT-4o (two-iteration agentic pipeline), and dZiner running its full design loop. AgentD requires an average of 9087 tokens per sample at a cost of $0.022, substantially lower than dZiner, which consumes 193,875 tokens and costs $0.533 per sample. Although AgentD's token usage is

higher than the direct prompting baseline (Vanilla GPT-4o), it remains far more cost-efficient than dZiner while providing a significantly richer, structured optimization process.

S10 Uncertainty in Prediction Tools

Accurately predicting protein–ligand binding affinity remains a significant challenge. In this work, we used BAPULM within the AgentD pipeline, while Boltz-2 outputs binding affinity alongside predicted 3D structures.[S10,S11] AutoDock Vina, T-Alpha, and Docking App RF are also widely used tools.[S12–S14] Although these methods enable rapid estimation, their predictive accuracy on standardized benchmarks such as CASF-2016 is limited, and reliable free-energy evaluation typically requires downstream refinement with molecular dynamics (MD) simulations. Table S7 summarizes the performance variability across commonly used affinity prediction models on the CASF-2016 benchmark, highlighting the inherent uncertainty in structure-based scoring. This evaluation reflects performance on a single dataset, making it difficult to generalize these accuracy levels. The results highlight the intrinsic challenges of binding affinity prediction—no single tool can fully replace MD-based free-energy evaluation.

Table S7: Performance of binding affinity prediction models on the CASF 2016 benchmark. Metrics reported are Pearson correlation coefficient (R^2), root-mean-square error (RMSE), and mean absolute error (MAE).

**Method**	R^2	**RMSE**	**MAE**
Bapulm	0.914 ± 0.004	0.898 ± 0.0172	0.645 ± 0.0166
T-Alpha	0.869	1.112	0.875
Docking App RF	0.83	1.38	1.13
AutoDock Vina	0.60	2.35	1.94

For ADMET prediction errors, the information is based on the Deep-PK publication. The table compares ADMET predictions from ADMETlab 2.0 with those from 3-fold cross-validated Deep-PK. Matthews Correlation Coefficient (MCC) is used for classification tasks, while R^2 is used for regression tasks. The table also indicates the property category and the

level of improvement observed with Deep-PK predictions.

Table S8: Comparison of ADMET predictions between ADMETlab 2.0 and 3-fold cross-validated Deep-PK, including average, standard deviation, metric, category, and improvement classification. Matthews Correlation Coefficient (MCC) is used for classification tasks, just as R^2 is used for regression tasks. The table is reconstructed from the Supplementary Information in the Deep-PK paper.[S15]

Name	ADMETlab 2.0	3-fold CV Deep-PK (avg)	3-fold CV Deep-PK (std)	Metric	Category	Improvement category
Drug metabolism (cyp1a2 substrate)	0.30	0.612	0.022	MCC	Metabolism	significant improvement
Drug metabolism (cyp2c19 substrate)	0.30	0.583	0.134	MCC	Metabolism	significant improvement
NR-AR Tox	0.35	0.779	0.02	MCC	Toxicity	significant improvement
NR-AR-LBD Tox	0.47	0.672	0.021	MCC	Toxicity	significant improvement
Ames	0.61	0.719	0.007	MCC	Toxicity	reasonable improvement
Drug metabolism (cyp2c9 substrate)	0.39	0.581	0.02	MCC	Metabolism	reasonable improvement
Oral bioavailability 20%	0.41	0.59	0.03	MCC	Absorption	reasonable improvement
Maximum recommended daily dose	0.47	0.623	0.012	MCC	Toxicity	reasonable improvement
Intestinal absorption	0.69	0.811	0.041	MCC	Absorption	reasonable improvement
NR-AhR Tox	0.57	0.694	0.017	MCC	Toxicity	reasonable improvement
NR-ER Tox	0.32	0.45	0.023	MCC	Toxicity	reasonable improvement
NR-ER-LBD Tox	0.36	0.519	0.029	MCC	Toxicity	reasonable improvement
NR-PPAR-gamma Tox	0.34	0.462	0.093	MCC	Toxicity	reasonable improvement
Drug efflux pump inhibitor (pgp_inhibitor)	0.72	0.837	0.012	MCC	Absorption	reasonable improvement
Drug efflux pump substrate (pgp_substrate)	0.54	0.739	0.007	MCC	Absorption	reasonable improvement
50% Growth inhibition to Tetraphymena pyriformis after 48 hours Tox	0.72	0.836	0.003	R2	Toxicity	reasonable improvement
Respiratory Tox	0.51	0.623	0.016	MCC	Toxicity	reasonable improvement
Skin sensitivity	0.46	0.587	0*	MCC	Toxicity	reasonable improvement
SR-ATAD5 Tox	0.36	0.468	0.015	MCC	Toxicity	reasonable improvement
SR-HSE Tox	0.39	0.549	0.011	MCC	Toxicity	reasonable improvement
SR-P53 Tox	0.37	0.505	0.026	MCC	Toxicity	reasonable improvement
BBB (logBB)	0.72	0.737	0.006	MCC	Distribution	no improvement
Bioconcentration factor Tox	0.79	0.781	0.005	R2	Toxicity	no improvement
Intestinal permeability (Caco2) logP	0.75	0.787	0.005	MCC	Absorption	no improvement
Carcinogenicity Tox	0.48	0.481	0.031	MCC	Toxicity	no improvement
Clearance	0.68	0.664	0.017	R2	Excretion	no improvement
Drug metabolism (cyp1a2 inhibitor)	0.70	0.746	0.006	MCC	Metabolism	no improvement
Drug metabolism (cyp2c19 inhibitor)	0.68	0.705	0.006	MCC	Metabolism	no improvement
Drug metabolism (cyp2c9 inhibitor)	0.67	0.663	0.009	MCC	Metabolism	no improvement
Drug metabolism (cyp2d6 inhibitor)	0.56	0.629	0.011	MCC	Metabolism	no improvement
Drug metabolism (cyp2d6 substrate)	0.55	0.647	0.025	MCC	Metabolism	no improvement
Drug metabolism (cyp3a4 inhibitor)	0.66	0.692	0.004	MCC	Metabolism	no improvement
Drug metabolism (cyp3a4 substrate)	0.44	0.444	0.012	MCC	Metabolism	no improvement
Liver Tox (dili)	0.79	0.841	0*	MCC	Toxicity	no improvement
Eye Tox (corrosion)	0.91	0.954	0*	MCC	Toxicity	no improvement
Eye Tox (irritation)	0.88	0.897	0.009	MCC	Toxicity	no improvement
Oral bioavailability 30%	0.58	0.498	0.014	MCC	Absorption	no improvement
Marine Tox (Fathead minnow)	0.75	0.767	0.002	R2	Toxicity	no improvement
Fraction unbound in human plasma	0.76	0.719	0.005	R2	Distribution	no improvement
Liver Tox (h_ht)	0.46	0.466	0.011	MCC	Toxicity	no improvement
Heart rhythm disorder	0.78	0.786	0.008	MCC	Toxicity	no improvement
Marine Tox (Daphnia magna)	0.52	0.565	0.018	R2	Toxicity	no improvement
logD (octanol-water distribution coefficient)	0.89	0.912	0.001	R2	property	no improvement
logP (octanol-water partition coefficient)	0.96	0.965	0*	R2	property	no improvement
logS (water solubility)	0.85	0.868	0.002	R2	property	no improvement
Intestinal permeability (MDCK)	0.73	0.773	0.006	R2	Absorption	no improvement
NR-Aromatase Tox	0.26	0.315	0.033	MCC	Toxicity	no improvement
Plasma protein binding	0.73	0.765	0.008	R2	Distribution	no improvement
Rat oral acute Tox	0.55	0.579	0.003	MCC	Toxicity	no improvement
SR-ARE Tox	0.47	0.552	0.01	MCC	Toxicity	no improvement
SR-MMP Tox	0.66	0.735	0.017	MCC	Toxicity	no improvement
Half-life of a drug	0.48	0.543	0.013	MCC	Excretion	no improvement
Volume distribution	0.78	0.702	0.011	R2	Distribution	no improvement

S11 Baseline Structural Modification

To assess whether the preference for polar functional-group edits observed in AgentD arises from an intrinsic language-model bias or from explicit property-aware guidance, we analyzed molecular refinements generated by a vanilla GPT-4o model without access to predicted ADMET properties, using the full set of 100 seed molecules across two successive refinement iterations.

Approximately 10% of vanilla GPT-4o outputs fall into the "analysis failed" category, corresponding to invalid or structurally inconsistent SMILES that cannot be reliably interpreted; this rate is substantially lower for AgentD with property-aware refinement, as shown in Figure 6f. In addition, a small fraction of cases exhibit no structural change, indicating that the model often preserves the original molecule rather than executing a concrete edit. Among the successfully modified cases, refinements are dominated by simple alkyl and halogen substitutions (Figure S9), highlighting the difficulty of producing chemically targeted and consistently interpretable transformations in the absence of explicit property-aware guidance.

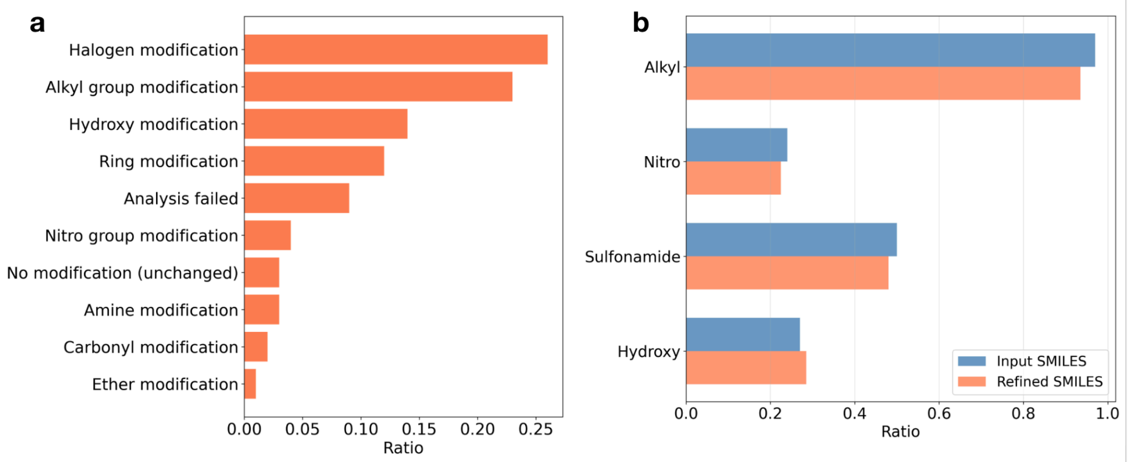

Figure S9: Vanilla GPT-4o molecular refinement analysis. **a** Fraction of molecules undergoing different modification types across 100 seed molecules over two refinement iterations. **b** Functional-group frequencies before refinement and after two iterations.

Overall, these observations show that vanilla GPT-4o primarily performs chemically

conservative substitutions, favoring alkyl and halogen edits without systematically steering molecular composition toward specific physicochemical objectives. In contrast, AgentD exhibits targeted polar functional-group modifications when guided by ADMET-derived weaknesses, indicating that the polar-edit preference observed in AgentD arises from property-aware optimization rather than intrinsic language-model bias.

S12 Strategy–Structure Alignment Analysis

To assess whether the molecular refinements produced by AgentD are aligned with its intended objectives, we performed a strategy–structure alignment analysis. For each refinement, we compared the intended modification strategy described in the model's natural-language rationale with the actual structural change observed in the updated SMILES. We analyzed 194 refinement steps arising from two successive LLM-based molecular refinement rounds, encompassing both seed-to-first and first-to-second refinement steps. Each event was classified based on the consistency between the intended modification strategy and the realized structural change, including perfect alignement, partial alignment and mismatch.

To determine alignment categories, we quantified strategy–structure consistency by comparing the intended edit described in the LLM rationale with the actual molecular change between the original and updated SMILES. Intended actions (add, remove, replace, modify, or no change) and target functional groups were extracted from the rationale using keyword and synonym matching. Actual structural changes were identified using RDKit by detecting differences in functional-group counts between input and output molecules based on curated SMARTS patterns. An alignment score in [0,1] was computed as the fraction of intended changes realized in the corresponding structural edit, with closely related groups treated as equivalent. Based on this score, refinements were categorized as perfect (score ≥ 0.8), partial ($0.4 \leq$ score < 0.8), or mismatch (score < 0.4).

When considering edited cases only, alignment ratios remain consistent across refinement

rounds. In the first refinement, 47.1% of edits are perfectly aligned, 32.9% partially aligned, and 20.0% mismatched; in the second refinement, these ratios are 46.9%, 31.3%, and 21.9%, respectively (Figure S10). This stability indicates that AgentD maintains comparable execution fidelity under iterative refinement.

Importantly, even when individual refinements exhibit partial alignment or localized mismatches, the collective refinement process yields systematic improvements in molecular quality, as reflected by upward shifts in QED distributions and increased compliance with empirical drug-likeness rules (Figure 6d–e). This indicates that strategy–structure deviations primarily reflect local execution constraints rather than a breakdown of chemically meaningful optimization at the molecule-population level.

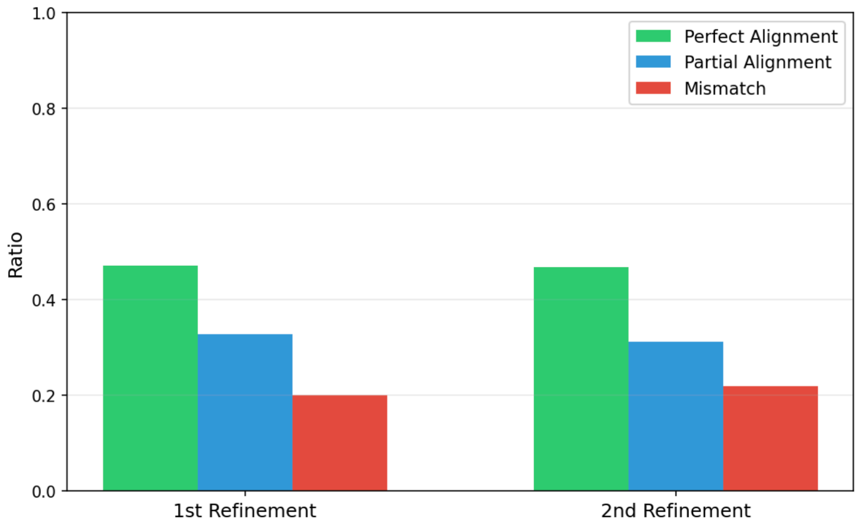

Figure S10: Strategy–structure alignment ratios across successive refinement rounds. Shown are the fractions of perfectly aligned, partially aligned, and mismatched refinements among edited cases for the 1st and 2nd refinement rounds.

References

(S1) Weller, S.; Toennießen, A.; Schaefer, B.; Beigl, T.; Muenchow, A.; Böpple, K.; Hofmann, U.; Gillissen, B. F.; Aulitzky, W. E.; Kopp, H.-G.; others The BCL-2 inhibitor ABT-199/venetoclax synergizes with proteasome inhibition via transactivation of the MCL-1 antagonist NOXA. *Cell death discovery* **2022**, *8*, 215.

(S2) Leonetti, A.; Sharma, S.; Minari, R.; Perego, P.; Giovannetti, E.; Tiseo, M. Resistance mechanisms to osimertinib in EGFR-mutated non-small cell lung cancer. *British Journal of Cancer* **2019**, *121*, 725–737.

(S3) Liu, Y.; Ott, M.; Goyal, N.; Du, J.; Joshi, M.; Chen, D.; Levy, O.; Lewis, M.; Zettlemoyer, L.; Stoyanov, V. RoBERTa: A robustly optimized BERT pretraining approach. **2019**,

(S4) Jhoti, H.; Williams, G.; Rees, D. C.; Murray, C. W. The 'rule of three' for fragment-based drug discovery: where are we now? *Nature Reviews Drug Discovery* **2013**, *12*, 644–644.

(S5) Lipinski, C. A.; Lombardo, F.; Dominy, B. W.; Feeney, P. J. Experimental and computational approaches to estimate solubility and permeability in drug discovery and development settings. *Advanced Drug Delivery Reviews* **2001**, *46*, 3–26.

(S6) Veber, D. F.; Johnson, S. R.; Cheng, H.-Y.; Smith, B. R.; Ward, K. W.; Kopple, K. D. Molecular properties that influence the oral bioavailability of drug candidates. *Journal of Medicinal Chemistry* **2002**, *45*, 2615–2623.

(S7) Ghose, A. K.; Viswanadhan, V. N.; Wendoloski, J. J. A knowledge-based approach in designing combinatorial or medicinal chemistry libraries for drug discovery. 1. A qualitative and quantitative characterization of known drug databases. *Journal of Combinatorial Chemistry* **1999**, *1*, 55–68.

(S8) Congreve, M.; Carr, R.; Murray, C.; Jhoti, H. A 'Rule of Three' for fragment-based lead discovery? *Drug Discovery Today* **2003**, *8*, 876–877.

(S9) Oprea, T. I.; Teague, S. J.; Seeber, G.; Wade, R. C. Is there a leadlike space? A critical assessment of lead-likeness criteria. *Journal of Chemical Information and Computer Sciences* **2001**, *41*, 1308–1320.

(S10) Meda, R. S.; Farimani, A. B. BAPULM: Binding Affinity Prediction using Language Models. 2024; `https://arxiv.org/abs/2411.04150`.

(S11) Passaro, S.; Corso, G.; Wohlwend, J.; Reveiz, M.; Thaler, S.; Somnath, V. R.; Getz, N.; Portnoi, T.; Roy, J.; Stark, H.; Kwabi-Addo, D.; Beaini, D.; Jaakkola, T.; Barzilay, R. Boltz-2: Towards Accurate and Efficient Binding Affinity Prediction. 2025.

(S12) Trott, O.; Olson, A. J. AutoDock Vina: Improving the speed and accuracy of docking with a new scoring function, efficient optimization, and multithreading. *Journal of Computational Chemistry* **2010**, *31*, 455–461.

(S13) Kyro, G. W.; Smaldone, A. M.; Shee, Y.; Xu, C.; Batista, V. S. T-ALPHA: A Hierarchical Transformer-Based Deep Neural Network for Protein–Ligand Binding Affinity Prediction with Uncertainty-Aware Self-Learning for Protein-Specific Alignment. *Journal of Chemical Information and Modeling* **2025**, *65*, 2395–2415, PMID: 39965912.

(S14) Macari, G.; Toti, D.; Pasquadibisceglie, A.; Polticelli, F. DockingApp RF: A State-of-the-Art Novel Scoring Function for Molecular Docking in a User-Friendly Interface to AutoDock Vina. *International Journal of Molecular Sciences* **2020**, *21*.

(S15) Myung, Y.; de Sá, A. G. C.; Ascher, D. B. Deep-PK: deep learning for small molecule pharmacokinetic and toxicity prediction. *Nucleic Acids Research* **2024**, *52*, W469–W475.
